# How wide is the window opened by high-resolution relaxometry on the internal dynamics of proteins in solution?

**DOI:** 10.1007/s10858-021-00361-1

**Published:** 2021-03-23

**Authors:** Albert A. Smith, Nicolas Bolik-Coulon, Matthias Ernst, Beat H. Meier, Fabien Ferrage

**Affiliations:** 1 Institut für Medizinische Physik und Biophysik, Universität Leipzig, Härtelstraße 16-18, 04107 Leipzig, Germany; 2 Physical Chemistry ETH Zurich, Vladimir-Prelog-Weg 2, 8093 Zurich, Switzerland; 3 Laboratoire des biomolécules, LBM, Département de Chimie, École normale superieure, PSL University, Sorbonne Université, CNRS, 75005 Paris, France

**Keywords:** Dynamics, Relaxometry, Detector analysis, Solution-state NMR

## Abstract

The dynamics of molecules in solution is usually quantified by the determination of timescale-specific amplitudes of motions. High-resolution nuclear magnetic resonance (NMR) relaxometry experiments—where the sample is transferred to low fields for longitudinal (*T*_1_) relaxation, and back to high field for detection with residue-specific resolution—seeks to increase the ability to distinguish the contributions from motion on timescales slower than a few nanoseconds. However, tumbling of a molecule in solution masks some of these motions. Therefore, we investigate to what extent relaxometry improves timescale resolution, using the “detector” analysis of dynamics. Here, we demonstrate improvements in the characterization of internal dynamics of methyl-bearing side chains by carbon-13 relaxometry in the small protein ubiquitin. We show that relaxometry data leads to better information about nanosecond motions as compared to high-field relaxation data only. Our calculations show that gains from relaxometry are greater with increasing correlation time of rotational diffusion.

**Supplementary Information:**

The online version contains supplementary material available at 10.1007/s10858-021-00361-1.

## Introduction

Understanding the molecular determinants of protein function requires not only the characterization of their structural properties but also of their dynamics. Nuclear magnetic resonance (NMR) is an ideal tool for detailed investigations of protein dynamics, due to a variety of high-resolution (residue-specific) experiments available that are sensitive to specific timescales of motion. In principle, this allows one to separate dynamics into specific ranges of correlation times. Then, the interpretation of dynamics data can be simplified by timescale separation, since different types of motion occur on different timescales (Frauenfelder et al. 1991; Henzler-Wildman et al. 2007).

High-field solution-state NMR provides quantitative, timescale-specific information on residue-specific dynamics, using a set of relaxation experiments typically measuring: longitudinal relaxation rate constants (*R*_1_ = 1/*T*_1_), cross-relaxation rate constants via nuclear Overhauser effect (NOE, *σ*_IS_), and transverse relaxation rate constants (e.g. *R*_2_ = 1/*T*_2_) or cross-correlated cross-relaxation rate constants, at one or several magnetic fields (Palmer 2004). High-resolution relaxometry (HRR) has been developed to increase the information about dynamics compared to existing high-field experiments (Chou et al. 2012; Redfield 2012; Charlier et al. 2013; Kiryutin et al. 2016). One polarizes an NMR sample at high field, transfers the sample shuttle to a chosen position in the stray field of the magnet with a lower field for *R*_1_ relaxation, and transfers the sample back to high field to obtain residue-specific resolution and sensitive detection (Clarkson et al. 2009; Redfield 2012; Charlier et al. 2013). A large range of magnetic fields is reached by simply varying the shuttling distance in the bore of the magnet, so that one may obtain site-specific nuclear magnetic resonance relaxation dispersion (NMRD) profiles using a single magnet. As currently implemented, fields ~ × 40 smaller than the field for detection are accessible (14 MHz vs. 600 MHz ^1^H frequency) for applications to proteins (Charlier et al. 2013; Cousin et al. 2018). Therefore, one may, in theory, characterize motions over timescales ~ × 30–40 slower. Much lower fields can be reached using a different setup, but with a sample shuttle system that is so far too slow for applications to biological macromolecules (Zhukov et al. 2018).

However, a problem remains in that regardless of experimental setup, tumbling of a molecule in solution masks motions significantly slower than the tumbling itself, potentially limiting the benefits of HRR. We would like to determine, in the presence of molecular tumbling, what is the range of correlation times of internal motion that we can determined with HRR, and whether it is broader than when high-field data only is used. Existing methods of data analysis may be limited in their ability to determine this: typically, multiple high-field or relaxometry data is analyzed using either spectral-density mapping (SDM) (Peng et al. 1992; Ishima et al. 1995; Kaderavek et al. 2016) and/or the model-free (MF) approach (Halle et al. 1981; Lipari et al. 1982; Clore et al. 1990; Halle 2009), although other approaches are also used (Tugarinov et al. 2001; Calandrini et al. 2010; Khan et al. 2015; Hsu et al. 2018). Results of SDM describe the combined motion of molecular tumbling in solution and the internal dynamics of the protein; the latter often are of greater interest, but difficult to characterize if convoluted with the tumbling. In principle, MF separates the internal protein dynamics from molecular tumbling, but the resulting parameters of dynamics can be biased, particularly when internal motions occur on a distribution of timescales (Smith et al. 2017). Then, whether using SDM or MF, it can be challenging to demonstrate that relaxometry improves the characterization of slow motions, especially because molecular tumbling masks motions significantly slower than the tumbling itself. Here, we use the detector approach to dynamics, which we have recently introduced in the context of solid-state NMR and later adapted to solution-state NMR (Smith et al. 2018, 2019a, b), to identify the ranges of correlation times probed by ensembles of relaxation rate constants collected with high-resolution relaxometry or high-field NMR alone.

## Theory

**Fig. 1:**
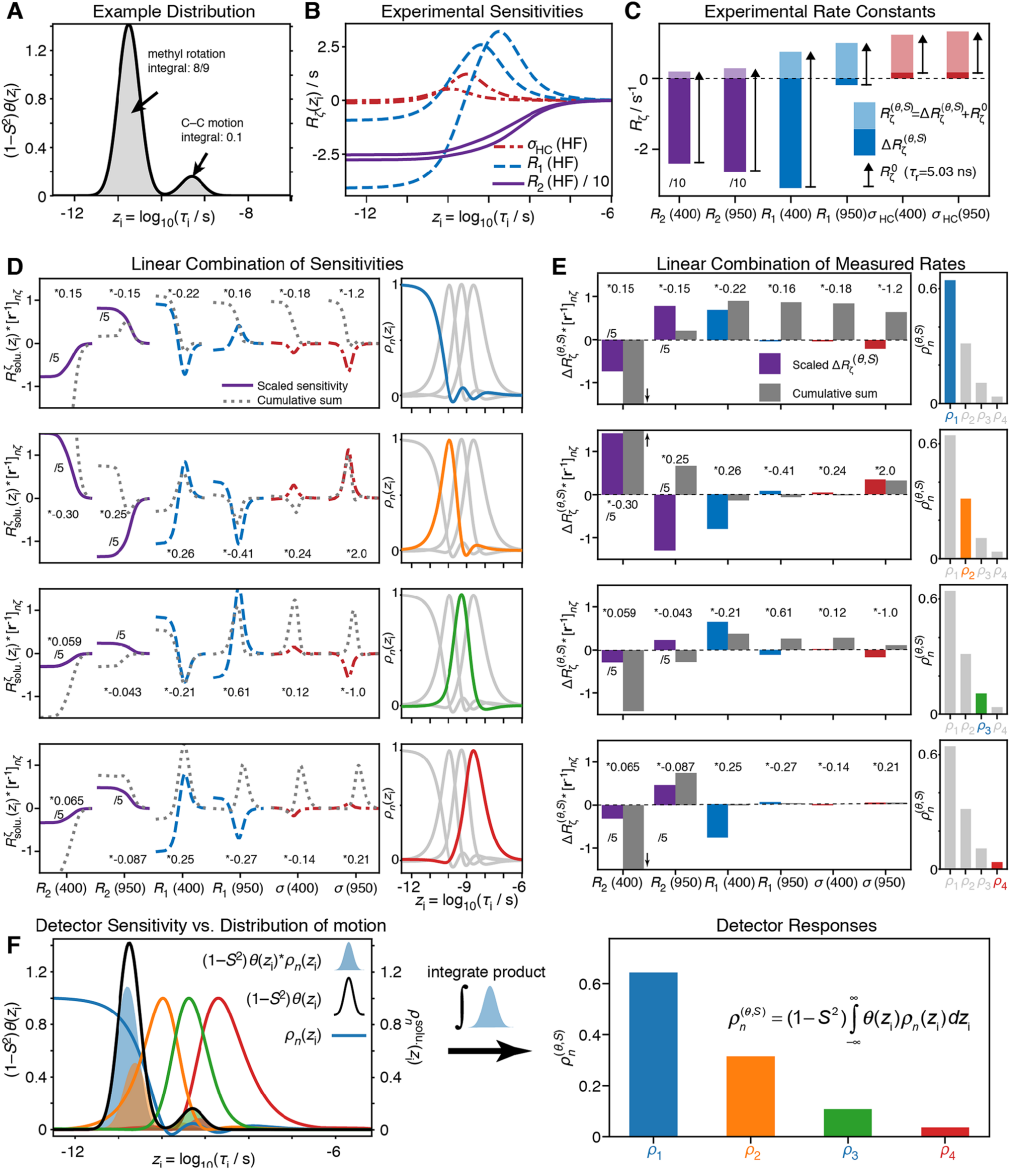
Constructing detectors from experimental data. **a** Shows a possible distribution of motion for a methyl group. We include a distribution of correlation times around 18 ps resulting from methyl rotation (where $(1 - S_{{{\text{met}}}}^{2} )$ for a tetrahedral geometry is 8/9), and a second distribution resulting from C–C bond motion at 500 ps, with an order parameter $S_{{{\text{CC}}}}^{2} = 0.1$, so that the integral of the distribution for this region is $S_{{{\text{met}}}}^{2} (1 - S_{{{\text{CC}}}}^{2} )$ = 0.1. **b** Sensitivities, $R_{\zeta } (z_{{\text{i}}} )$, of 6 experiments (*R*_2_, *R*_1_, *σ*_HC_ at 400 and 950 MHz) and **c** rate constants for these experiments, calculated using the distribution in **a**. In **c**, the total of the rate constants, $R_{\zeta }^{(\theta ,S)}$, is positive and shown as a lighter color, where the total is broken into contributions from internal motion ($\Delta R_{\zeta }^{(\theta ,S)}$, darker bars, may be negative) and tumbling motion ($R_{\zeta }^{0}$, black arrows). *R*_*2*_ experiments are shown in purple (displayed at 1/10 their true size), *R*_1_ in blue, and *σ*_HC_ in red. In each row in **d**, we construct each of four detectors, $\rho_{n}$, by taking the experimental sensitivities, scaling them (colored) and adding them together, yielding the sensitivities shown on the right. Grey lines show the cumulative sum of the current sensitivity and sensitivities to the left. In **e**, the same operation is performed on the rates $\Delta R_{\zeta }^{(\theta ,S)}$, to yield the detector responses, $\rho_{n}^{(\theta ,S)}$, where colored bars are the scaled $\Delta R_{\zeta }^{(\theta ,S)}$, and grey bars give the cumulative sum of rate constants to the left of the bar (*R*_2_ sensitivities and rate constants are displayed at 1/5 their true size; cumulative sum is shown full size). The result is that each detector response is equal to the overlap integral of the distribution of motion (**f**, left, black line) and the detector sensitivity (colored lines, where the product is shown as a colored, shaded area). The results of the integrals are the detector responses (**f**, right), which are equal to the scaled sum of experimental rate constants

The detector approach to dynamics analysis allows one to characterize the amount of motion for well-defined ranges of correlation times, making it a suitable method to determine if relaxometry can be used to better characterize slower internal motions. Detector analysis yields several detector responses, each of which characterizes the amplitude of motion in a well-defined window of correlation times, known as the sensitivity (e.g. Fig. 1b, c). One begins with experimental sensitivities corresponding to relaxation data measured at a given static magnetic field, which quantify how much relaxation is induced by a given motion as a function of correlation time. Linear combinations of experimental sensitivities yield detector sensitivities, for which the linear combinations are optimized to cover different ranges of correlation times. Then, the range of correlation times covered by a set of detector sensitivities depends directly on the sensitivity of the experiments used to generate those detectors. In the absence of molecular tumbling, we would find that using HRR down to fields of 14 MHz allow us to resolve different timescales via detectors for motions about × 30–40 slower than would be accessible based on high-field data only (lowest high field used here is 600 MHz). However, molecular tumbling masks some of these motions. By taking into account the correlation time of the tumbling when calculating experimental sensitivities, we are able to predict the extent to which using relaxometry data improves resolution of slower motions, as we will discuss below.

The original detector approach was targeted primarily at the analysis of solid-state NMR data, where it is straightforward to define experimental sensitivities (Smith et al. 2018). On the other hand, in solution-state NMR, stochastic motions that lead to relaxation originate from internal motions in the molecular frame and overall rotational diffusion of the entire molecule. We have recently defined a sensitivity to *internal* motion in the presence of overall motion, which allows us to separate these two contributions (Smith et al. 2019a). Note that detectors, as well as most other approaches, such as model-free (Lipari et al. 1982) and two-step (Halle et al. 1981) approaches assumes statistical independence of internal motion and rotational diffusion; other approaches such as the slowly relaxing local structure (SRLS) model do not use this assumption (Polnaszek et al. 1975; Tugarinov et al. 2001). Statistical independence is generally assumed in case two motions are separated in timescale, via the adiabatic approximation (Halle 2009). However, motions with similar correlation times may also be statistically independent; for molecular tumbling, we would expect that an internal motion does not influence molecular tumbling (and vice versa) if it does not cause large distortions in the molecular shape. In particular, in the presence of isotropic molecular tumbling, statistical independence should allow us to factorize the correlation function and characterize motions as slow (and slightly slower) than the tumbling itself. In the presence of overall rotational diffusion with a correlation time, $\tau_{{\text{r}}}$, the relaxation rate constant, $R_{\zeta }^{(\theta ,S)}$ for a measurement at a given magnetic field (denoted *ζ*) is given by:

1 $$\begin{gathered} R_{\zeta }^{(\theta ,S)} = R_{\zeta }^{0} + \Delta R_{\zeta }^{(\theta ,S)} \hfill \\ \Delta R_{\zeta }^{(\theta ,S)} = (1 - S^{2} )\int\limits_{ - \infty }^{\infty } {\theta (z_{i} )R_{\zeta }^{solu.} (z_{i} )dz_{i} } . \hfill \\ \end{gathered}$$

Here, $R_{\zeta }^{0}$ is the relaxation rate constant obtained if the tumbling molecule is internally rigid ($\tau_{{\text{r}}}$ must be known to calculate $R_{\zeta }^{0}$). $\Delta R_{\zeta }^{(\theta ,S)}$ is the difference between $R_{\zeta }^{0}$ and the actual rate constant, where the difference results from internal motion. The internal motion is described by the generalized squared order parameter, *S*^2^, and $\theta (z_{{\text{i}}} )$, which describes how that motion is distributed as a function of correlation time. $z_{{\text{i}}}$ is defined as the log-correlation time, $z_{{\text{i}}} = \log_{10} (\tau_{{\text{i}}} /1{\text{ s}})$. The distribution of motion, $(1 - S^{2} )\theta (z_{{\text{i}}} )$, may describe a continuum of correlation times or a discrete distribution of correlation times. In general, the correlation function for internal motion is then given by

2 $$C_{i} (t) = S^{2} + (1 - S^{2} )\int\limits_{ - \infty }^{\infty } {\theta (z_{i} )\exp \left( { - t/(10^{{z_{i} }} \cdot 1 \, s)} \right)dz_{i} } ,$$

where $\theta (z_{{\text{i}}} )$ integrates to one. In case the motion is described by a few discrete correlation times,

3 $$\begin{gathered} (1 - S^{2} )\theta (z_{i} ) = (1 - S^{2} )\sum\limits_{n} {A_{n} \delta (z_{i} - z_{n} )} \hfill \\ \sum\limits_{n} {A_{n} } = 1 \hfill \\ C_{i} (t) = S^{2} + (1 - S^{2} )\sum\limits_{n} {A_{n} \exp \left( { - t/(10^{{z_{n} }} \cdot 1 \, s)} \right)} \hfill \\ \end{gathered}$$

By describing the correlation function with a distribution, we assume very little about the correlation function—only that it consists of decaying exponential terms, but with a few or many discrete correlation times, or alternatively a continuum of correlation times. By limiting assumptions on the form of $C_{{\text{i}}} (t)$, we eliminate a major source of bias in the data analysis (see Smith et al. 2017, 2018, 2019a) for details).

The change in a relaxation-rate constant due to internal motion, $\Delta R_{\zeta }^{(\theta ,S)}$, may be negative or positive: internal motion attenuates the effective size of anisotropic NMR interactions (the sizes of the anisotropy for an interaction without motion δ and after scaling by internal motion δ_eff_ are related as $\delta_{{{\text{eff}}}}^{2} = S^{2} \cdot \delta^{2}$), reducing the relaxation induced by molecular tumbling. However, internal motion may also induce relaxation directly, i.e., increasing the relaxation rate constant. These two contributions add together to yield the total value of $\Delta R_{\zeta }^{(\theta ,S)}$.

In Eq. (1), $R_{\zeta }^{{{\text{solu}}{.}}} (z_{{\text{i}}} )$ is the solution-state sensitivity for internal motion, which defines how an internal motion with a given correlation time $\left( {\tau_{{\text{i}}} = 10^{{z_{{\text{i}}} }} \cdot 1{\text{ s}}} \right)$ contributes to $\Delta R_{\zeta }^{(\theta ,S)}$. Examples of $R_{\zeta }^{{{\text{solu}}{.}}} (z_{{\text{i}}} )$ are given in Fig. 1b, for a molecule tumbling with a correlation time of $\tau_{{\text{r}}}$ = 5.03 ns, where sensitivities are calculated for the relaxation of a ^13^C nucleus in a ^13^CD_2_H methyl group. Note that solution-state sensitivities are always negative for very short correlation times: as discussed, internal motion attenuates the effective size of anisotropic interactions, reducing the relaxation due to molecular tumbling. Since very fast motion induces very little relaxation directly, the net effect is negative. Slower internal motion ($\tau_{{\text{i}}}$ > 1 ps) induces some relaxation directly, so that sensitivities become larger and, in some cases, positive. However, all sensitivities approach zero for correlation times significantly longer than the rotational correlation time for overall rotational diffusion, since molecular tumbling in solution masks the motions significantly slower than the tumbling. The shape of the sensitivity is indicative of the range of correlation times to which a given experiment provides information.

### Distributions to detectors: an example

To better understand the information in the detector analysis, we give a concrete example to describe how detectors are built and dynamics characterized. Let us consider a methyl group, rotating with a distribution of correlation times with a maximum at 18 ps. In addition, the whole methyl group reorients (C–C axis motion) with a distribution of correlation times with a maximum at 500 ps. The methyl rotation has an order parameter of $S_{{{\text{met}}}}^{2} = 1/9$ (corresponding to tetrahedral geometry, $[\tfrac{1}{2}(3\cos^{2} (109.47^{^\circ } ) - 1)]^{2} = 1/9$), and the order parameter for the C–C axis motion is $S_{{{\text{CC}}}}^{2} = 0.1$, such that $S_{{{\text{met}}}}^{2} (1 - S_{{{\text{CC}}}}^{2} ) = 0.1$(faster motions reduce the contributions of the slower motions). The resulting distribution of correlation times for internal motion, $(1 - S^{2} )\theta (z_{{\text{i}}} )$, is shown in Fig. 1a. Then, the term $\Delta R_{\zeta }^{(\theta ,S)}$ (for a given relaxation rate at a given magnetic field represented by the subscript *ζ*) is the result of integrating the product of the sensitivity of the experiment, $R_{\zeta }^{{{\text{solu}}{.}}} (z_{{\text{i}}} )$, with $(1 - S^{2} )\theta (z_{{\text{i}}} )$ (Eq. (1)). Here, we consider 6 measurements: the three relaxation-rate constants *R*_1_, *R*_2_, and *σ*_ΗC_ (NOE) measured at two magnetic fields 400 and 950 MHz. The sensitivities to internal motion are shown in Fig. 1b, and the result of the overlap integral, $\Delta R_{\zeta }^{(\theta ,S)}$, is shown as dark-colored bars in Fig. 1c. Note that $\Delta R_{\zeta }^{(\theta ,S)}$ itself often is negative, however, the measured rate constant, $R_{\zeta }^{(\theta ,S)}$, is the sum of $\Delta R_{\zeta }^{(\theta ,S)}$ and the contribution from molecular tumbling ($R_{\zeta }^{0}$, arrows in Fig. 1c, calculated for $\tau_{r} = 5.03{\text{ ns}}$), where the total must always be positive, resulting in the light-colored bars in Fig. 1c.

When performing an experimental characterization of dynamics, we do not know the distribution of motion, $(1 - S^{2} )\theta (z_{{\text{i}}} )$, but instead have $R_{\zeta }^{(\theta ,S)}$ for a series of experiments (indexed with $\zeta$). The first step of the detectors analysis is, for each experiment, to subtract the contribution to the rate constant from molecular tumbling, yielding $\Delta R_{\zeta }^{(\theta ,S)}$, where $\Delta R_{\zeta }^{(\theta ,S)}$ provides information about internal motion. An example of that information can be seen in Fig. 1c, where we obtain a negative $\Delta R_{\zeta }^{(\theta ,S)}$ for *R*_1_ at 950 MHz. This indicates that motions with correlation times where the sensitivity of this experiment is negative make a greater contribution to $\Delta R_{\zeta }^{(\theta ,S)}$ than do motions having correlation times where the sensitivity is positive. Indeed, in Fig. 1b, the sensitivity crosses zero at about 100 ps, showing us that there is more contribution to relaxation from motion at correlation times shorter than ~ 100 ps than above 100 ps. This is consistent with our assumed distribution, having methyl rotation with a correlation time around 18 ps $\left( {\left( {1 - S_{{{\text{met}}}}^{2} } \right) = 8/9} \right)$. However, because the sensitivity of any single experiment is non-zero over a broad range of correlation times, it makes separation of timescales difficult without further information. Sensitivities of two or more experiments with different functional forms can be linearly combined to yield detectors. Detectors are defined as functions that are non-zero only over a narrow range of correlation times. We have previously denoted this linear combination as:

4 $$\rho_{n}^{{{\text{solu}}{.}}} (z_{{\text{i}}} ) = \sum\limits_{\zeta } {[{\mathbf{r}}^{ - 1} ]_{n,\zeta } R_{\zeta }^{{{\text{solu}}{.}}} (z_{{\text{i}}} )} .$$

Details of optimization of the linear combination, and the corresponding notation, are given in (Smith et al. 2018, 2019a), where we point out here that ${\mathbf{r}}$ is a matrix used for fitting rate constants to detector responses, and ${\mathbf{r}}^{ - 1}$ is its pseudoinverse. In Fig. 1d, we show how the experimental sensitivities for our example (Fig. 1b) may be combined to yield four different optimized detector sensitivities, $\rho_{n}^{{{\text{solu}}{.}}} (z_{{\text{i}}} )$.

Finally, we want to obtain each detector response, $\rho_{n}^{(\theta ,S)}$, which describes the amount of motion in the sensitivity window defined by the detector $\rho_{n}^{{{\text{solu}}{.}}} (z_{{\text{i}}} )$. We start by summing the experimental terms $\Delta R_{\zeta }^{(\theta ,S)} (z_{{\text{i}}} )$, using the same weighting as in Eq. (4), yielding

5 $$\rho_{n}^{(\theta ,S)} = \sum\limits_{\zeta } {[{\mathbf{r}}^{ - 1} ]_{n,\zeta } \Delta R_{\zeta }^{(\theta ,S)} } .$$

We may insert the expression for $\Delta R_{\zeta }^{(\theta ,S)}$ found in Eq. (1) to obtain the following relationship:

6 $$\begin{aligned} \rho_{n}^{(\theta ,S)}  = \sum\limits_{\zeta } {[{\mathbf{r}}^{ - 1} ]_{n,\zeta } (1 - S^{2} )\int\limits_{ - \infty }^{\infty } {\theta (z_{{\text{i}}} )R_{\zeta }^{{{\text{solu}}{.}}} (z_{{\text{i}}} )dz_{{\text{i}}} } } \\ = (1 - S^{2} )\int\limits_{ - \infty }^{\infty } {\theta (z_{{\text{i}}} )} \underbrace {{\sum\limits_{\zeta } {[{\mathbf{r}}^{ - 1} ]_{n,\zeta } R_{\zeta }^{{{\text{solu}}{.}}} (z_{{\text{i}}} )} }}_{{\rho_{n}^{\text{solu.}} (z_{{\text{i}}} )}}dz_{{\text{i}}} \\ \rho_{n}^{(\theta ,S)} = (1 - S^{2} )\int\limits_{ - \infty }^{\infty } {\theta (z_{{\text{i}}} )} \rho_{n}^{{{\text{solu}}{.}}} (z_{{\text{i}}} )dz_{{\text{i}}} . \\ \end{aligned}$$

We must swap the order of the summation and the integral, and then insert Eq. (4), so that we finally find that the detector response, $\rho_{n}^{(\theta ,S)}$, calculated via linear combination of experimental data, are equal to the overlap integral of the distribution of motion and the detector sensitivity. In practice, the detector responses are obtained from a linear fit rather than a simple linear combination and one renormalizes the data depending on its standard deviation, allowing us to account for variations in data quality and to enforce restrictions on the allowed values of the detector responses (Smith et al. 2018).

To demonstrate the equality—that is, to show that the linear combination of the experimentally determined $\Delta R_{\zeta }^{(\theta ,S)}$ is in fact equal to the overlap of the detector sensitivity with the distribution of motion (Eq. (6))—we take the terms $\Delta R_{\zeta }^{(\theta ,S)}$ and sum them according to Eq. (5), with the results in Fig. 1e (right). Then, we find that we may also obtain the same detector responses by calculating the integral of the product of $(1 - S^{2} )\theta (z_{{\text{i}}} )$ and each detector sensitivity, $\rho_{n} (z_{{\text{i}}} )$, in Fig. 1f. The product is shown for each example detector in Fig. 1f (left) as a colored, shaded region, and the resulting integrals of the shaded regions are shown in Fig. 1f (right). We see from the large, blue shaded region, and somewhat smaller orange shaded region that *ρ*_1_ and *ρ*_2_ overlap the large peak in $(1 - S^{2} )\theta (z_{{\text{i}}} )$, corresponding to methyl rotation, and therefore characterize this part of the overall dynamics. *ρ*_3_ and *ρ*_4_ (green and red shaded regions) overlap the smaller peak (C–C motion). As expected, the result is the same as obtained using the sum of experimental data. Here, we apply this principle to better understand the information content of relaxometry data, and to analyze the dynamics of Isoleucine δ1 methyl groups in Ubiquitin.

## Results

### Detector sensitivities of high-field and relaxometry data

**Fig. 2:**
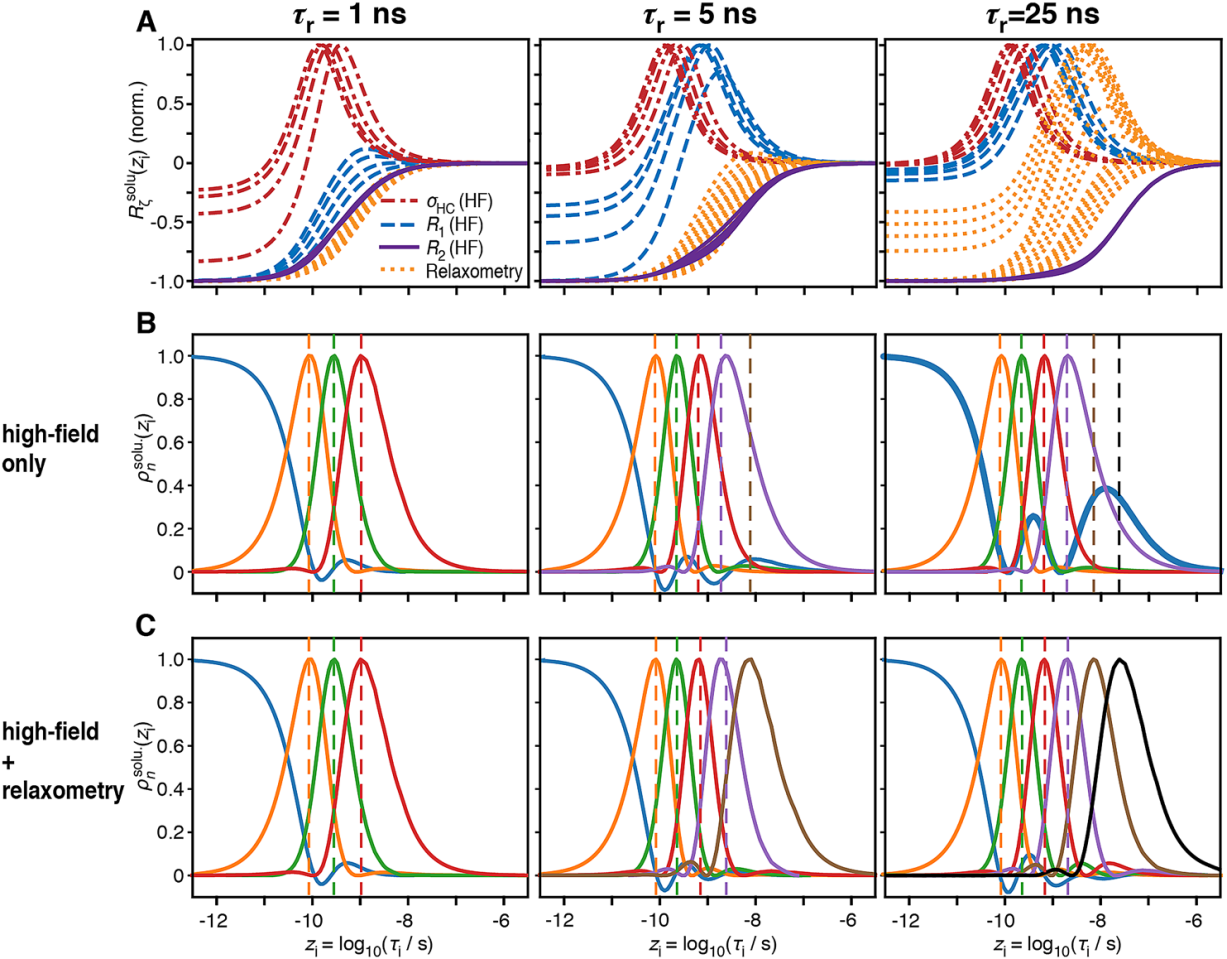
Experimental and detector sensitivities for molecular tumbling at three different correlation times ($\tau_{{\text{r}}}$ = 1, 5, 25 ns). In **a**, we calculate the normalized sensitivities for a set of experiments measuring ^13^C relaxation of a ^13^CD_2_H-labeled methyl group using high-field (400, 600, 800 and 950 MHz) and relaxometry data (20 *R*_1_ experiments between 13.5 and 170 MHz). Dashed blue lines show high-field *R*_1_ and dashed/dotted red lines show the dipolar cross-relaxation rate (*σ*_HC_), solid purple lines show high-field *R*_2_, and dotted orange lines show relaxometry data. **b** Detector sensitivities optimized from high-field data only, where different colors are used only to distinguish the different detectors. **c** Sensitivities using all data. For comparison of detector position, dotted lines in **b** indicate the positions of the detector maxima from **c**, and the dotted lines in **c** indicate detector maxima from **b.** In **b**, right, the first detector is highlighted (bold, blue) to show the unusual shape obtained when using only high-field data to characterize motion of a large molecule. The number of detectors is chosen from the size of singular values obtained during detector optimization (discussed below)

In the previous section, we showed that it was possible to obtain detector sensitivities from optimized linear combinations of experimental sensitivities to internal motion of the protein. If the center of a detector sensitivity, $\rho_{n}^{{{\text{solu}}{.}}} (z_{{\text{i}}} )$, is shifted to longer correlation times, that detector is able to better characterize slower motions with the corresponding detector responses. If we obtain more and narrower detectors at long correlation times, we can describe slow motions with better resolution. However, the sensitivities obtained for a set of detectors depends on the sensitivity of the experimental rate constants to internal motion, and this in turn depends on the correlation time of the molecular tumbling. Therefore, we begin with a theoretical investigation of the effect of molecular tumbling, where we optimize the sets of detector sensitivities for three different correlation times for tumbling. For each correlation time of molecular tumbling, we optimize detectors using either only high-field data or high-field data combined with HRR data. Experimental sensitivities are shown in Fig. 2a, for three correlation times for molecular tumbling $\tau_{{\text{r}}}$ = 1 ns, 5 ns, and 25 ns.

For each value of $\tau_{{\text{r}}}$, a set of detectors is optimized from the experimental sensitivities either without (Fig. 2b) or with (Fig. 2c) the relaxometry data. When optimizing detectors without relaxometry (high-field data set), we include longitudinal *R*_1_, transverse *R*_2_, and dipolar cross-relaxation rates *σ*_HC_ at fields of 400, 600, 800, and 950 MHz, yielding a total of 12 experiments. When including relaxometry, the 12 high-field experiments are considered with an additional 20 *R*_1_ relaxometry experiments with fields in the range of 13.5–170 MHz. Note that the number of detectors is chosen to limit the error of the resulting detector responses: we previously demonstrated how to optimize detectors using singular value decomposition (Smith et al. 2019a), and demonstrated that if *N* detectors are used to analyse a data set, then the error depends on the inverse of the largest *N* singular values obtained during detector optimization. Therefore, we may define a threshold for the inverse singular values, choosing *N* so that all of the *N* inverse singular values remain below the threshold. In SI Sect. 3, we use the experimental data to determine a reasonable threshold for the singular values (resulting in a value of 0.1). This threshold is applied when determining the number of detectors to use in Figs. 2b, c and 4. The value of the threshold is marked in Fig. 5.

For a small molecule ($\tau_{{\text{r}}}$ = 1 ns), we see that detector sensitivities obtained with and without relaxometry are almost identical (Fig. 2b/c, left). The sensitivities of the relaxometry data are similar to that of *R*_2_, so that we have little to gain from relaxometry in terms of measuring slower motion. For a small protein with a correlation time for molecular tumbling $\tau_{{\text{r}}}$ = 5 ns (middle), the sensitivities of relaxometry data are shifted to slightly longer correlation times in the low nanosecond range. This shift of detectors towards longer correlation times with relaxometry predicts the possibility to better characterize motions in the low nanosecond range. For larger proteins, for instance with $\tau_{{\text{r}}}$ = 25 ns (right), detector sensitivities obtained with relaxometry extend to much lower correlation times, and we see that the relaxometry data (Fig. 2a, right) has sensitivities extending to significantly longer correlation times. On the other hand, we obtain an unusual shape for one of the detectors obtained with high-field data (Fig. 2b, right, bold blue line). Non-zero sensitivity of this detector both at short correlation times ($\tau_{{\text{i}}}$ < 30 ps) and longer correlation times (local maximum at 13 ns) indicates that the experimental data cannot unambiguously separate motions at these timescales.

This problem arises because none of the experimental data is sufficiently different at these two ranges of correlation times to distinguish the motion: *R*_2_ is uniformly sensitive to fast motion due to attenuation of couplings, resulting in reduction of relaxation from molecular tumbling. This contribution is eventually canceled out for longer correlation times (normalized sensitivity reaches − 0.5 at 25 ns, or approximately $\tau_{{\text{r}}}$), where the slower motion makes contributions to *J*(0). On the other hand, high-field sensitivities of the rate constants *σ*_HC_ and *R*_1_ are most sensitive in the range of 33 ps<$\tau_{{\text{i}}}$<6 ns  (i.e. having a sensitivity at least 0.5x the corresponding maximum sensitivity). Comparing these ranges of correlation times, we see that there isn’t enough difference in the high-field *R*_2_, *R*_1_, and *σ*_HC_ sensitivities for the ranges $\tau_{{\text{i}}}$ < 30 ps and 6 ns < $\tau_{{\text{i}}}$ < 25 ns to produce an unambiguous detector sensitivity. Thus, the resulting detector is sensitive at short correlation times, but has an additional hump around 13 ns. When we only have high-field data, we cannot differentiate these two timescale regimes; responses in *ρ*_1_ in this case can be due to fast motion, slow motion, or a combination of both motions. However, if relaxometry data is available, we can directly sample the timescale window between ~ 6 and ~ 25 ns and therefore obtain separated detector windows (Fig.2c, right). When fitting the spectral density function with a small number of correlations times (e.g. with model-free or other approaches) to the same set of experiments, the same ambiguity exists. Consider that one typically tries to find a fit that uses the minimum number of parameters. In many cases, restricting the number of parameters can lead to a unique best fit. Of course, this does not guarantee that the true distribution of motion is not more complex than determined by such a fit. If more parameters were allowed, one would find that the fit is not unique at all. Detectors, on the other hand, allow for an arbitrary number of motions present. Not knowing the number of motions will result in greater ambiguity in the characterization of dynamics, which becomes apparent when optimizing detector sensitivities. This ambiguity may not appear at all when fitting data using a given model with a minimum number of parameters.

**Fig. 3:**
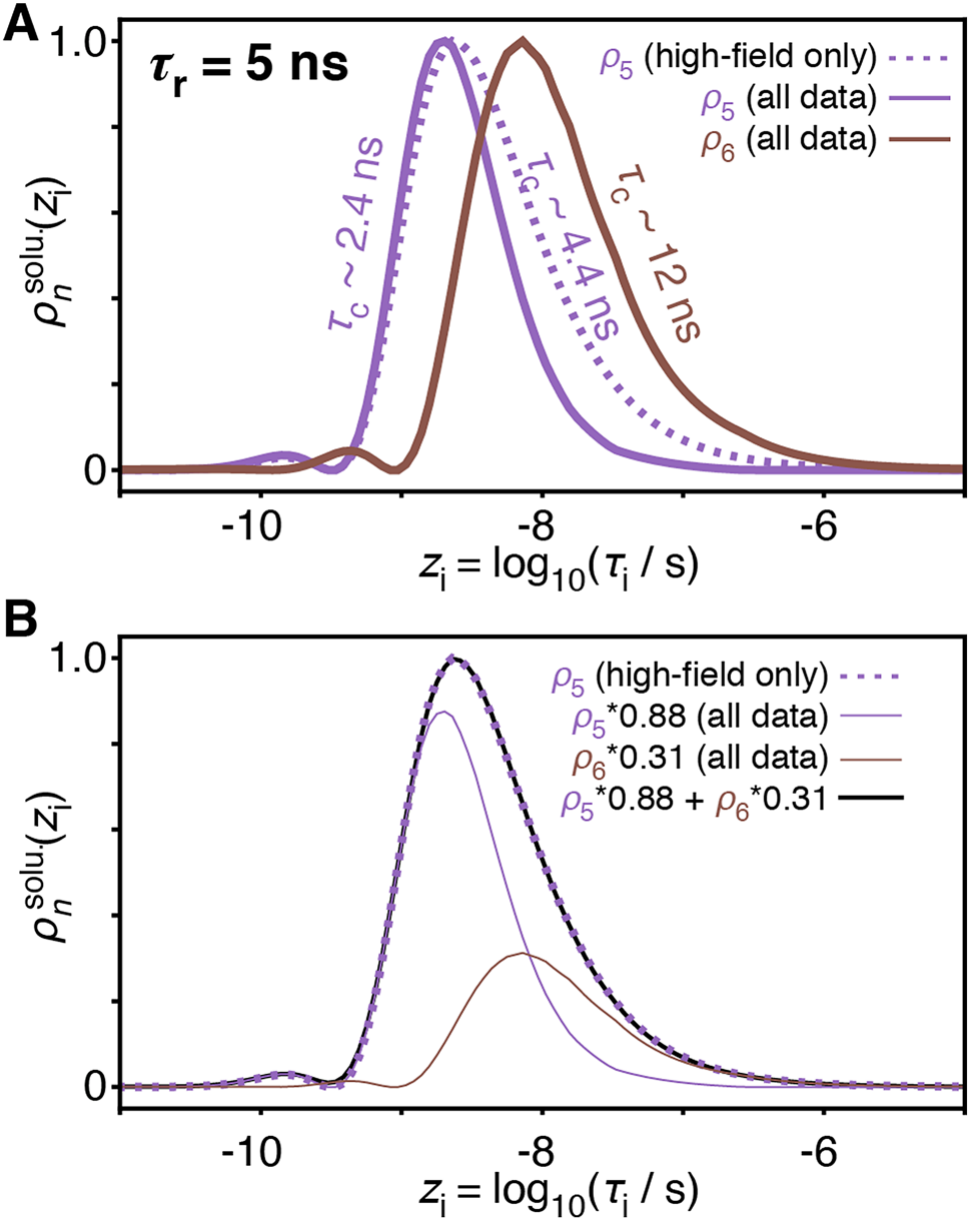
Comparing detectors sensitive to slow motion. **a** Sensitivity of the detector *ρ*_5_ for high-field relaxation data only (purple, dashed line), and sensitivities of *ρ*_5_ and *ρ*_6_ for the combination of high-field and relaxometry data (purple and brown, solid lines), obtained for $\tau_{{\text{r}}}$ = 5 ns (see Fig. 2b, c, middle). **b** The detector $\rho_{5}^{{{\text{solu}}{.}}} (z_{{\text{i}}} )$ obtained for high-field data only is approximately equal to a linear combination of $\rho_{5}^{{{\text{solu}}{.}}} (z_{{\text{i}}} )$ and $\rho_{6}^{{{\text{solu}}{.}}} (z_{{\text{i}}} )$ for the full data set, showing that relaxometry enhances the resolution of the sensitivity to slow motion

Relaxation rates at low field enhance the resolution of motions in the low nanosecond range. Relaxometry experiments are not generally sensitive to motions significantly slower than can be observed via transverse relaxation at high field (Fig. 2a). For example, consider the behavior of the transverse relaxation rate constant sensitivities in Fig. 2a for $\tau_{{\text{r}}}$ = 5 ns and 25 ns. The sensitivities of relaxometry and transverse relaxation rate constants extend to equally long correlation times for $\tau_{{\text{r}}}$ = 5 ns, and *R*_2_ is sensitive to slightly longer correlation times than the relaxometry experiments for $\tau_{{\text{r}}}$ = 25 ns (although using lower fringe fields could extend relaxometry sensitivity further). Therefore, the resulting detectors should, in principle, be able to identify similarly slow motion with or without relaxometry. For $\tau_{{\text{r}}}$ = 5 ns, the first four detectors are nearly the same using only high-field data (Fig. 2b, middle) or relaxometry and high-field data (Fig. 2c, middle). With relaxometry data, we obtain two additional detectors, vs. only one more detector than when only using high-field data. These are shown in more detail in Fig. 3a. At a glance, it appears that *ρ*_6_ obtained using relaxometry is sensitive to motions significantly slower than can be measured with *ρ*_5_ using high-field data only. Upon closer inspection, we find that the sensitivity of *ρ*_5_ obtained for the high-field data set can actually be split to yield *ρ*_5_ and *ρ*_6_ of the full data set, as illustrated in Fig. 3b. *ρ*_6_ of the full data set is not sensitive to motions slower than observed with *ρ*_5_ of the high-field data; instead, *ρ*_5_ and *ρ*_6_ of the full data set simply describe slow motions with higher resolution, allowing us to better determine the timescale of such motions. This additional resolution is critical in estimating correlation times: if, for a given residue, we only detect motion with the detector that is sensitive to the longest correlation times (e.g. *ρ*_5_ for the high-field data set), then we have very little information regarding its true correlation time. The reason is that the measured response may be due to motion near the center of the detector’s sensitivity, but it may also be due to significantly slower motion with higher amplitude (which is then partially masked by the molecular tumbling). Relaxometry data leads to enhanced detector resolution, which allows one to better estimate correlation times of slow motions.

Increased resolution also offers an advantage when considering anisotropic molecular tumbling. When separating molecular tumbling from internal protein motion, the relative orientations of the residual NMR interaction tensors and the molecular tumbling tensor influence the contribution that the tumbling makes to the overall motion. This effect is important if molecular tumbling is not fully isotropic (i.e. the molecule is not spherical). If one can reasonably estimate the direction and shape of the residual tensor resulting from internal motion, one may correctly account for the influence of the anisotropy of tumbling when separating molecular tumbling and internal motion (for both model-free analysis, or for detector analysis, although the latter method is not published). For side-chain dynamics in this study, however, it is not possible to estimate the residual tensor without knowing the populations of all rotamers, so that we are instead forced to assume isotropic tumbling, although we expect slightly anisotropic molecular tumbling (Tjandra et al. 1995). Deviations of molecular tumbling from full isotropy may result in artifactual contributions to the detector sensitive to the longest correlation times: *ρ*_5_ when only high-field data is used, or *ρ*_6_ when using high-field and relaxometry data (see Fig. 3). In this case, motion on the nanosecond timescale identified with *ρ*_5_ using relaxometry can be clearly separated from molecular tumbling whereas *ρ*_5_ of the high-field only data set may include both internal motions and the effect of deviation from isotropic molecular tumbling.

Relaxometry, in principle, provides us with the ability to resolve much longer correlation times, where the lowest field used here (13.5 MHz) should be able to provide information on motions approximately 30 times slower than the lowest high field data (400 MHz). However, the experimental sensitivities and detector optimization indicate that we only achieve those gains if molecular tumbling is slow enough. That is, the slow motions observable with relaxometry are masked by the tumbling. So for $\tau_{{\text{r}}}$ = 1 ns, no gains are made, but for $\tau_{{\text{r}}}$ = 25 ns, we obtain clear benefits from the relaxometry data, potentially improving characterization of much slower motion, resolving ambiguity between faster and slower motions in the nanosecond regime, and helping separate true internal motion from deleterious effects due to anisotropic molecular tumbling.

### Experimental data analysis

The theoretical analysis demonstrates that, in principle, relaxometry should improve the resolution with which we can describe slow motion. We now treat an experimental data set to evaluate in practice how much benefit we can obtain from relaxometry. We analyze methyl dynamics of all isoleucine residues in ubiquitin with and without relaxometry (data analyzed in this article is found in its Supplementary Information, originally from refs. Cousin et al. 2018; Kaderavek et al. 2019), where we use the ICARUS-corrected data here (Charlier et al. 2013; Bolik-Coulon et al. 2020)). An additional fit of the relaxometry data only can be found in SI Fig. 4. For this sample, the molecular tumbling correlation time was previously determined to be 5.03 ns, from an analysis of backbone ^15^N relaxation data with the program ROTDIF (Berlin et al. 2013), so that modest gains are expected from the relaxometry data (Fig. 2). Note that definition of the sensitivities for ^13^C relaxation in a methyl group is complicated by the fact that the H–C and D–C dipole tensor correlation function is different than that for the ^13^C chemical shift anisotropy; see SI section 2 for details.

**Fig. 4:**
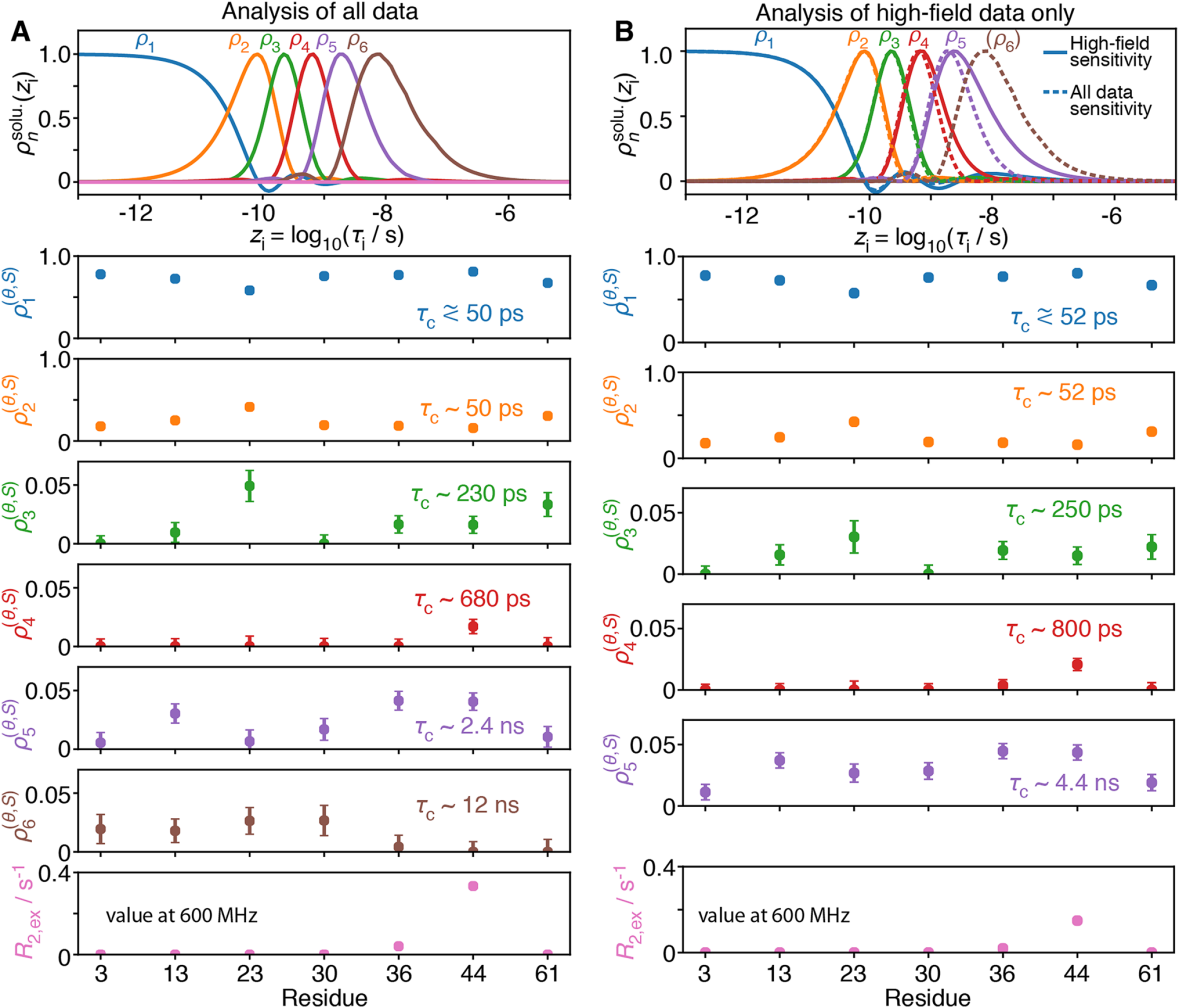
Detector analysis of isoleucine methyl dynamics in ubiquitin, for all 7 Ile in the molecule, using high-field data with and without low-field relaxation data (^13^C relaxation for a ^13^CD_2_H group). In each plot, residue-specific detector responses are plotted with sensitivities shown at the top (analysis performed assuming $\tau_{r}$ = 5.03 ns). **a** Detector responses using a combination of high-field data (*R*_1_, *R*_2_, *σ*_HC_ at 400, 600, 800, 950 MHz) and relaxometry data (*R*_1_ at 13.5–390 MHz). **b** Responses from a high-field only data set. Note that the detector sensitivities are shifted to shorter correlation times for the high-field data set, as can be seen in the top plot in **b** (solid lines: high-field only, dashed lines: full relaxometry data set). Data fits can be found for **a** in SI Fig. 5, and for **b** in SI Fig. 6, as well as in SI Tables S3–S7

**Fig. 5:**
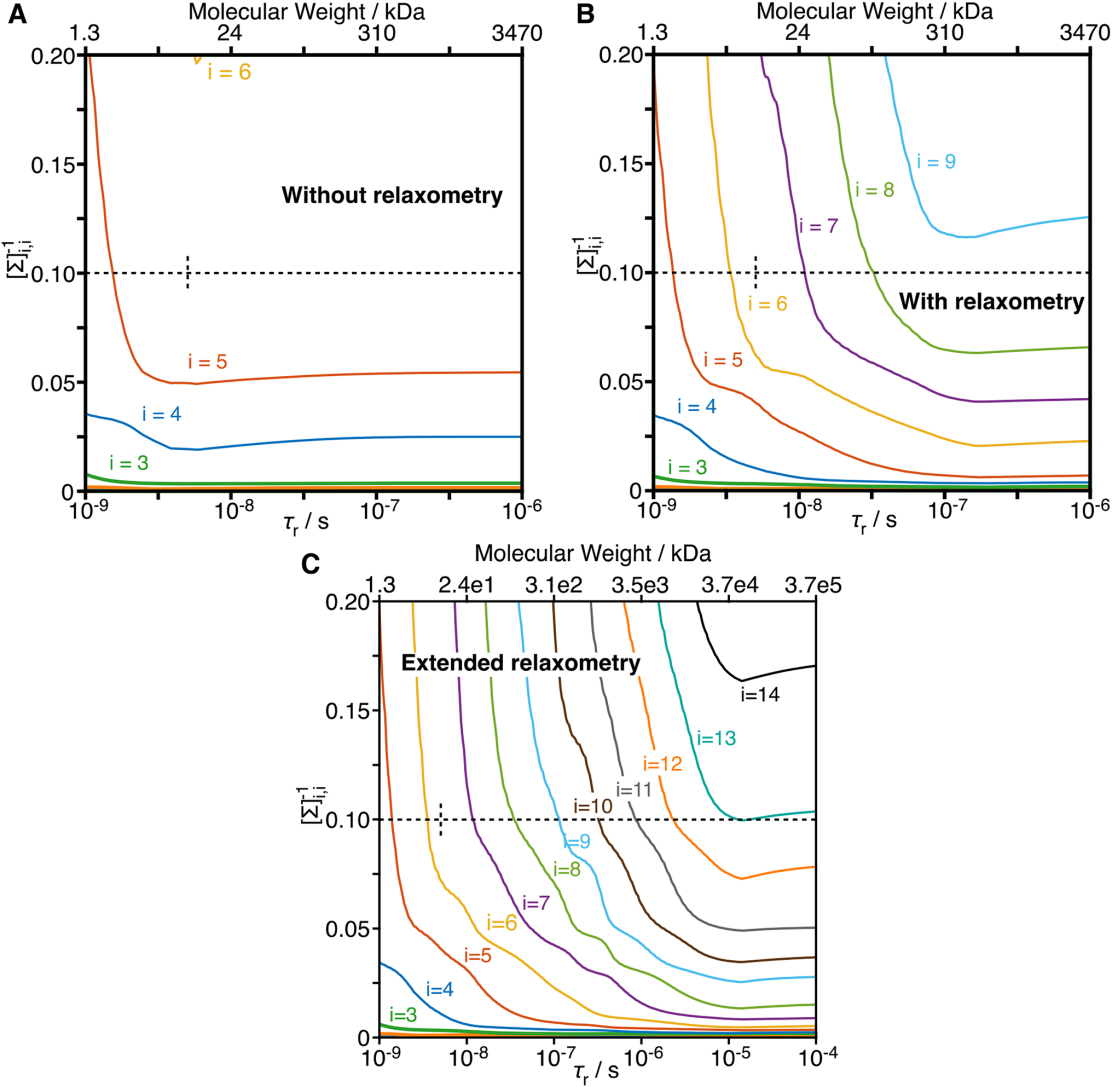
Inverse of the singular values as a function of rotational correlation time. Singular value decomposition was performed on a matrix containing the sensitivities of a set of experiments (without and with relaxometry), with normalization by the standard deviation of the measurement (for **a** and **b**, the experimental standard deviations were used. In **c**, additional experiments for which there is not experimental data, the median standard deviation of the relaxometry experiments was used). **a** Inverse of singular values as a function of $\tau_{{\text{r}}}$ for a data set without relaxometry (*R*_1_, *σ*_NH_, *R*_2_ at 400, 600, 800, 950 MHz). **b** Inverse of singular values with relaxometry using current technology (the set of magnetic fields is the same as in Fig. 2). **c** Result using extended relaxometry, where 25 additional fields log-spaced between 12 MHz and 400 kHz proton resonance frequency are included. The top axes provide rough estimates of molecular weights corresponding to the correlation times shown on the bottom axes, calculated with Stokes’ law, assuming a water viscosity of 8.9 × 10^–4^ Pa*s, a protein density of 1.37 g/mL, temperature of 298 K, and hydration layer of 3.2 Å (Cavanagh et al. 2007). Dashed lines show the threshold used to select the number of detectors (0.1) and the rotational correlation time for the data set used in this study ($\tau_{{\text{r}}}$ = 5.03 ns)

We have optimized detector sensitivities to analyze methyl-relaxation data obtained using high-field and relaxometry data (Fig. 4a). We obtain reasonable confidence intervals using 6 detectors for the full relaxometry data set, where the detector sensitive to the longest correlation times has a center that falls at about 12 ns (7 detectors yields higher resolution, but with higher standard deviation). The choice of number of detectors is discussed in SI section 3, and we will see below that the number of detectors is related to the size of singular values obtained during the detector optimization process (Fig. 5). In parallel, we have optimized detector sensitivities using only the high-field data (Fig. 4b); to obtain similar standard deviation on the resulting detectors, we use 5 instead of 6 detectors to analyze the high-field data set. Additional analysis of the high-field data set with 6 detectors can be found in SI Fig. 3.

We note two significant gains from the inclusion of relaxometry data. First, the center of the slowest detector moves from about 4.4 ns for the high-field data set to 12 ns for the full relaxometry data set, a result of the higher resolution provided by using relaxometry. Second, we have gained an additional data point, having 6 detectors instead of 5. If we analyze the high-field data with 6 detectors (SI Fig. 3), we find a significant increase in the standard deviations of the resulting detectors. These increases are especially pronounced for detectors sensitive to long correlation times, as expected, since relaxometry provides information on slower motions.

The detectors approach sheds light on the model-free analysis of ^13^C relaxation rate constants in methyl groups (Cousin et al. 2018; Kaderavek et al. 2019). We have previously shown that the analysis of relaxometry in Ile44 with a model-free approach adapted to methyl groups provided an effective correlation time for slow motions of the methyl axis $\tau_{{\text{s}}}$ = 1.3 ns, in agreement with the analyses shown in Fig. 4a. Non-zero detector responses for *ρ*_4_ (center ~ 680 ps) and *ρ*_5_ (2.4 ns) of the full data set, and non-zero responses for *ρ*_4_ (center ~ 800 ps) and *ρ*_5_ (center ~ 4.4 ns) of the high-field data set both point towards a mean correlation time in between these ranges (although we note that the responses may also be a results of a distribution of correlation times around ~ 1 ns). The high-field data set alone identifies a slow motion in the low nanosecond for Ile44. In contrast, we find that slower motions observed on Ile13 and Ile36 could not be precisely defined from an analysis of high-field relaxation only (Kaderavek et al. 2019). For these residues, we obtain a significant detector response for *ρ*_5_ of the high-field data, but no response on *ρ*_4_. The response on *ρ*_5_ alone is ambiguous at several levels: first one cannot determine whether a relatively low amplitude motion towards the center of the sensitivity of *ρ*_5_ results in the observed detector responses, or if the response results from a higher amplitude motion at a much longer correlation time (or some combination of these cases). In addition, we previously noted that because ubiquitin is not perfectly spherical, and we cannot reliably estimate the residual tensors from internal motion, we may have some artifactual contribution from tumbling in the detector which is sensitive to the longest correlation times (*ρ*_5_ for the high-field data set, and *ρ*_6_ for high-field and relaxometry data). Indeed, this seems a likely scenario; when considering only the high-field data, there is some motion detected with $\rho_{5}^{(\theta ,S)}$ for all residues. Thus, it would be difficult to assign with certainty to slightly higher sensitivities in $\rho_{5}^{(\theta ,S)}$ for Ile13 and Ile36 to nanosecond motions from high-field relaxation rates only.

On the other hand, if we consider the full data set, including relaxometry, we obtain an additional detector response, so that we have two detectors in the nanosecond range: *ρ*_5_ (2.4 ns) and *ρ*_6_ (12 ns). We find a larger detector response for *ρ*_5_ than *ρ*_6_ for both Ile13 and Ile36, which suggests that the observed motion is in fact found between 2.4 and 12 ns. This is in good agreement with the previous model-free analysis (Cousin et al. 2018), where effective correlation times of 3.1 ns and 2.5 ns were found for Ile13 and Ile36 respectively. The detector approach thus confirms the presence of motion in the low nanosecond range in three isoleucine side chains, by providing higher timescale resolution than high-field data alone. In addition, this enhanced resolution, obtained using relaxometry data, allows us to clearly distinguish internal motion in the low nanosecond range for Ile13, Ile36, and Ile44 from possible artifactual contributions from tumbling (whereas low responses now in $\rho_{6}^{(\theta ,S)}$ may capture some tumbling motion). The detector approach shows that, in two out of three cases, high-field relaxation rates do not contain sufficient resolution in the nanosecond regime to fully characterize the methyl dynamics and thus demonstrates that high-resolution relaxometry provides unique information.

### Quantifying information content: singular value decomposition

Information content of a data set, in particular when considering slow motion, depends on whether we have experiments sensitive to long correlation times (as provided by relaxometry), and whether tumbling of the molecule in solution masks slow motion. We have previously shown that detector accuracy is determined by a linear combination of the inverse of the singular values, obtained during detector optimization (using singular value decomposition, see (Smith et al. 2019a)). Then, the number of inverse singular values $\left( {[\Sigma ]_{i,i}^{ - 1} } \right)$ that fall below a chosen threshold determines the number of separate detectors that can be obtained from a data set. The choice of the threshold depends on how much error can be tolerated (here the threshold is taken to be 0.1; see SI section 3, where we discuss requirements for the threshold).

We plot the inverse singular values $[\Sigma ]_{i,i}^{ - 1}$ as a function of the tumbling correlation time for three data sets in Fig. 5. In Fig. 5a, we take only the set of high-field data used in Fig. 2a. Between $\tau_{{\text{r}}}$ = 1 ns and 3 ns (10^–9^ and 10^–8.5^ s), $[\Sigma ]_{4,4}^{ - 1}$ becomes marginally smaller and $[\Sigma ]_{5,5}^{ - 1}$ decreases significantly. After this point, little further improvement is observed: while the increase of the rotational correlation time unmasks slower motions, experiments in the high-field data set cannot characterize these slower motions better, mostly because slower nanosecond motions and very fast low picosecond motions are difficult to disentangle (see Fig. 2b for $\tau_{{\text{r}}}$ = 25 ns).

In contrast, if we include the magnetic fields used in relaxometry experiments (down to 13.5 MHz or 0.3 T), we see decreases in singular values up to $\tau_{{\text{r}}}$ ~ 100 ns (Fig. 5b). In this case, the increasing $\tau_{{\text{r}}}$ unmasks more motion, *and* we have the experimental data to characterize that motion. We still eventually reach a plateau, since the relaxometry experiments are sensitive to motions about 30 times slower than the high-field relaxation (*R*_1_ at 400 MHz vs. 13.5 MHz). Adding further relaxometry experiments at even lower fields causes the plateaus to occur at even longer correlation times, which we demonstrate by including fields as low as 400 kHz or 9 mT (Fig. 5c). Such low fields would, in principle, allow analysis with up to ~ 10 detectors, if the molecule tumbles sufficiently slowly, To our knowledge, quantitative, high-resolution NMR of ^13^C^1^H^2^H_2_ methyl groups in proteins has been demonstrated for molecular weights up to 360 kDa but not beyond yet (Rennella et al. 2015).

## Conclusions

High-resolution relaxometry provides information about motion with longer correlation times, which cannot be properly described with the lower resolution available from high-field relaxation alone. However, the amount of information available depends both on the availability of relaxometry data at low fields and on the correlation time of molecular tumbling of the molecule. For moderate-to-slow molecular tumbling, relaxometry is a useful addition to high-field relaxation data to characterize slower motion in the nanosecond range. In particular, for slowly tumbling macromolecules (for methyl dynamics, the tumbling correlation time is τ_r_ ~ 25 ns or more) high-field relaxation data alone cannot distinguish the fastest and slowest motions, so that relaxometry becomes a critical component of dynamics studies of large molecules. The detector approach highlights the need for relaxometry data for the analysis of picosecond and nanosecond motions in large macromolecules. In addition, even on a small protein as ubiquitin, which tumbles with τ_r_ = 5.03 ns, the detector approach demonstrates why methyl dynamics is better defined when high-field data is complemented with relaxometry measurements. Further improvements in relaxometry experiments, such as improving the sensitivity of the probes, and the ability to reach lower magnetic fields will make studies of larger molecules possible, where relaxometry becomes critical to characterize the picosecond and nanosecond range of motion, including slow functional motions of proteins in solution.

## Supplementary Information

Below is the link to the electronic supplementary material.

Supplementary file1 (PDF 2267 KB)
