# Supplementary material for: How wide is the window opened by high-resolution relaxometry on the internal dynamics of proteins in solution?
Source: J Biomol NMR. 2021 Mar 23;75(2):119–31. doi: 10.1007/s10858-021-00361-1 (PMC8018934; doi:10.1007/s10858-021-00361-1)
Supplement: Supplementary file 1 — Supplementary file1 (PDF 2267 KB) [file 10858_2021_361_MOESM1_ESM.pdf]

Supplementary information for:

How wide is the window opened by high-resolution relaxometry on the internal dynamics of proteins in solution?

Albert A. Smith[1,2]*, Nicolas Bolik-Coulon[3], Matthias Ernst[2], Beat H. Meier[2], Fabien Ferrage[3]*

[1]*Institut für Medizinische Physik und Biophysik, Universität Leipzig, Härtelstraße 16-18, 04107 Leipzig, Germany*

[2]*Physical Chemistry ETH Zurich, Vladimir-Prelog-Weg 2, 8093 Zurich, Switzerland*

[3]*Laboratoire des biomolécules, LBM, Département de chimie, École normale supérieure, PSL University, Sorbonne Université, CNRS, 75005 Paris, France.*

E-mail: *albert.smith-penzel@medizin.uni-leipzig.de*
Fabien.Ferrage@ens.fr

1. Calculation of relaxation rate constants for methyl groups

We report here the analytical expression of the carbon longitudinal (R_1), transverse (R_2) relaxation rates, as well as the ^{13}C-^{1}H cross-relaxation rate (σ_{CH}) in an isolated $C^1H^2H_2$ methyl group (Cousin et al. 2018):

$$
\begin{aligned}
R_1(^{13}\mathrm{C}) &= \frac{1}{3}\Delta\sigma_\mathrm{C}^2\Delta\omega_\mathrm{C}^2 J_\mathrm{CSA}(\omega_\mathrm{C}) + \frac{1}{4}d_\mathrm{CH}^2\left(J_\mathrm{CH}(\omega_\mathrm{H}-\omega_\mathrm{C}) + 3J_\mathrm{CH}(\omega_\mathrm{C}) + 6J_\mathrm{CH}(\omega_\mathrm{H}+\omega_\mathrm{C})\right) + \\
&\quad \frac{4}{3}d_\mathrm{CD}^2\left(J_\mathrm{CD}(\omega_\mathrm{H}-\omega_\mathrm{D}) + 3J_\mathrm{CD}(\omega_\mathrm{D}) + 6J_\mathrm{CD}(\omega_\mathrm{H}+\omega_\mathrm{D})\right), \\
R_2(^{13}\mathrm{C}) &= \frac{1}{18}\Delta\sigma_\mathrm{C}^2\Delta\omega_\mathrm{C}^2\left(4J_\mathrm{CSA}(0) + 3J_\mathrm{CSA}(\omega_\mathrm{C})\right) \\
&\quad + \frac{1}{8}d_\mathrm{CH}^2\left(4J_\mathrm{CH}(0) + J_\mathrm{CH}(\omega_\mathrm{H}-\omega_\mathrm{C}) + 3J_\mathrm{CH}(\omega_\mathrm{C}) + 6J_\mathrm{CH}(\omega_\mathrm{H}) + 6J_\mathrm{CH}(\omega_\mathrm{H}+\omega_\mathrm{C})\right) + \\
&\quad + \frac{2}{3}d_\mathrm{CD}^2\left(4J_\mathrm{CD}(0) + J_\mathrm{CD}(\omega_\mathrm{H}-\omega_\mathrm{D}) + 3J_\mathrm{CD}(\omega_\mathrm{D}) + 6J_\mathrm{CD}(\omega_\mathrm{H}) + 6J_\mathrm{CD}(\omega_\mathrm{H}+\omega_\mathrm{D})\right), \\
\sigma_\mathrm{HC} &= \frac{1}{4}d_\mathrm{CH}^2\left(-J_\mathrm{CH}(\omega_\mathrm{H}-\omega_\mathrm{C}) + 6J_\mathrm{CH}(\omega_\mathrm{H}+\omega_\mathrm{C})\right),
\end{aligned}
\tag{S1}
$$

where $d_{AB} = -(\mu_0/4\pi)\hbar\gamma_A\gamma_B r_{AB}^{-3}$, μ_0 is the permittivity of free space, $\hbar$ is Planck's constant divided by 2π, γ_A,γ_B are the gyromagnetic ratios of the two nuclei, r_{AB} is the distance between nuclei A and B ($r_\mathrm{CH} = 111.5$ pm, $r_\mathrm{DH} = 111.0$ pm), $\Delta\sigma_\mathrm{C}$ is the ^{13}C chemical shift anisotropy (CSA), and $\omega_\mathrm{C,H,D} = -\gamma_\mathrm{C,H,D}B_0$ is the Larmor frequency at magnetic field B_0, for ^{13}C, ^{1}H, or ^{2}H respectively. Note that we do not include the contribution to relaxation from neighbouring deuteriums (outside the methyl group), as these nuclei were only initially

considered to reproduce proton relaxation rates (Bolik-Coulon et al. 2020). We see in SI Fig. 1 that contributions from neighbouring deuterium to the carbon longitudinal and transverse relaxation are negligible over the range of magnetic fields considered here. These rates were calculated using the following spectral density function:

$$J_i(\omega) = \frac{2}{5}\Bigg[S^2_{\mathrm{met}}(\theta_i)\left(S^2_{\mathrm{f}} S^2_{\mathrm{s}} \frac{\tau_{\mathrm{r}}}{1+(\omega\tau_{\mathrm{r}})^2} + (1-S^2_{\mathrm{f}})\frac{\tau'_{\mathrm{f}}}{1+(\omega\tau'_{\mathrm{f}})^2} + S^2_{\mathrm{f}}(1-S^2_{\mathrm{s}})\frac{\tau'_{\mathrm{s}}}{1+(\omega\tau'_{\mathrm{s}})^2} \right) \\ (1-S^2_{\mathrm{met}}(\theta_i))\left(S^2_{\mathrm{f}} S^2_{\mathrm{s}} \frac{\tau'_{\mathrm{met}}}{1+(\omega\tau'_{\mathrm{met}})^2} + (1-S^2_{\mathrm{f}})\frac{\tau''_{\mathrm{f}}}{1+(\omega\tau''_{\mathrm{f}})^2} \right) + S^2_{\mathrm{f}}(1-S^2_{\mathrm{s}})\frac{\tau''_{\mathrm{s}}}{1+(\omega\tau''_{\mathrm{s}})^2} \Bigg] \quad \text{(S2)}$$

where τ_{r} is the global tumbling correlation time, S^2_{f} and S^2_{s} are the squared order parameters for fast and slow internal motions with correlation times τ_{f} and τ_{s}, respectively. Effective correlation times are defined as:

$$(\tau'_k)^{-1} = \tau_{\mathrm{r}}^{-1} + \tau_k^{-1},\ \ k = \{\mathrm{f,s,met}\} \\ (\tau''_k)^{-1} = \tau_{\mathrm{r}}^{-1} + \tau_{\mathrm{met}}^{-1} + \tau_k^{-1},\ \ k = \{\mathrm{f,s}\} \quad \text{(S3)}$$

where τ_{met} is the methyl rotation correlation time associated with squared order parameter:

$$S^2_{\mathrm{met}}(\theta_i) = (P_2(\cos\theta_i))^2. \quad \text{(S4)}$$

θ_i is the angle between the CC bond and the interaction *i*, where $P_2(x)$ indicates the second order Legendre polynomial defined as:

$$P_2(x) = \frac{3x^2-1}{2}. \quad \text{(S5)}$$

Here, $\theta_{\mathrm{CH}} = \theta_{\mathrm{CD}} = 109.47^\circ$ and $\theta_{\mathrm{CSA}} = 0^\circ$.

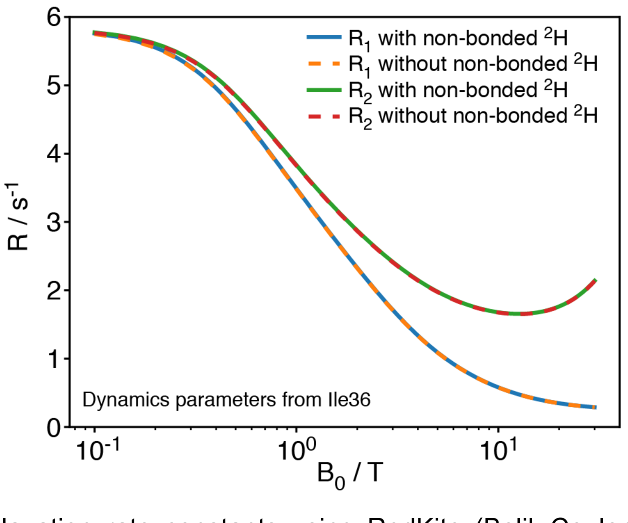

SI Fig. 1. Calculation of relaxation rate constants using RedKite (Bolik-Coulon et al. 2020) and ICARUS (Charlier et al. 2013, Bolik-Coulon et al. 2020) with and without inclusion of nearby (non-bonded) deuterium atoms as a function of magnetic field. Parameters used correspond to ICARUS fits of Ile36 relaxation, where

non-bonded deuterium was included in the model (Cousin et al. 2018): S_f^2 = 0.67; S_s^2 = 0.58; τ_r = 5.03 ns; τ_s = 2.48 ns; τ_f = 82.8 ps; τ_{met} = 8.05 ps; and $\Delta\sigma_c$ = 29 ppm.

Then, using the detector analysis, we limit assumptions about the form of the correlation function. We assume that the correlation function of internal motion, $C_I(t)$, is described by a distribution of motion, such that

$$C_I(t) = S^2 + (1-S^2)\int_{-\infty}^{\infty} \theta(z_i)\exp\left(-t/(10^{z_i}\cdot 1\text{ s})\right)dz_i,, \tag{S6}$$

and the total correlation function is

$$\begin{aligned} C(t) &= C_O(t)C_I(t) \\ C_O(t) &= \frac{1}{5}\exp(-t/\tau_r) \end{aligned}, \tag{S7}$$

The generality of this correlation function avoids a number of distortions that may arise when applying a more specific model, for example, a sum of several decaying exponentials (Smith et al. 2018, Smith et al. 2019).

However, we note that in eq. (S1), there are actually three different spectral densities, $J_{CH}(\omega)$, $J_{CD}(\omega)$, and $J_{CSA}(\omega)$: the spectral densities for reorientational motion of the H–C and D–C bonds, and reorientational motion of the ^{13}C CSA, respectively. It is reasonable to assume that $J_{CH}(\omega) = J_{CD}(\omega)$, since both bonds will be reoriented by methyl rotation around the C–C bond axis, and by reorientation of the C–C bond itself. On the other hand, the ^{13}C CSA tensor is considered to be parallel to the C–C bond, and therefore CSA-induced relaxation is not influenced by fast methyl rotation around the C–C bond. Then, the correlation functions of internal motion of the H–C and H–D dipole tensors and the ^{13}C CSA tensor are

$$\begin{aligned} C_{CH}(t) &= C_{CD}(t) = C_{met}(t)\cdot C_{CC}(t) \\ C_{CSA}(t) &= C_{CC}(t) \end{aligned}. \tag{S8}$$

where $C_{met}(t)$ is the correlation function for methyl rotation, and $C_{CC}(t)$ is the correlation function for C–C bond motion (we assume these motions are statistically independent). Then, for long times

$$C_{CH}(t) = C_{CD}(t) = S_{met}^2(\theta_i)\cdot C_{CC}(t)\ . \tag{S9}$$

$S_{met}^2(\theta_i)$ is calculated from the angle, θ_i, between of the principle axis of the dipole interaction and the C–C bond as $S_{met}^2(\theta_i) = [(3\cos^2\theta_i - 1)/2]^2$, which, if we take to be the

tetrahedral angle, 109.47°, we obtain $S^2_{\mathrm{met}}(\theta_i)=1/9$. We discuss how to resolve the differences in the correlation functions for detector analysis in the next section.

2. Treatment of methyl rotation using detectors

The presence of methyl rotation creates some challenges for analysis with detectors. Detector analysis assumes that all relaxation for a given nucleus results from the same correlation function. However, this is not true when methyl rotation is present; relaxation is induced by both reorientation of the chemical shift anisotropy tensor, which is parallel with the methyl C–C bond, and by reorientation of the dipole tensors, which are parallel to the H–C and D–C bonds. Then, methyl rotation has a significantly different influence on the two interactions: the order parameter for each interaction due to the rotation is obtained by calculating $S^2_{\mathrm{met}}(\theta_i)=[(3\cos^2\theta_i-1)/2]^2$. For the dipole tensors, this is the tetrahedral angle, 109.47°, but for the CSA, the angle is 0. Then, the corresponding order parameters are 1/9 and 1, respectively. Then, methyl rotation causes the correlation function of the dipole couplings to decay to 1/9, but has no influence on the correlation function of the CSA.

Suppose we only had relaxation due to internal motions that exclude the methyl rotation, but still had the scaling of dipole couplings due to the methyl rotation. For many of our experiments, this is actually the case because relaxation is acquired at low fields and therefore the relaxation is not very sensitive to the fast methyl rotation. For these experiments, a simple solution exists: we take the desired CSA value ($\Delta\sigma=25$ ppm) and multiply it by 3 when calculating the experimental sensitivities. Then, we may treat the experimental data as if both the dipole and CSA correlation functions have an initial reduction from 1 to $S^2_m=1/9$ (noting that CSA relaxation is proportional to $\Delta\sigma^2$, so scaling by 3 yields the desired factor of 9 to cancel out the factor of 1/9).

However, such a solution apparently fails when we consider relaxation due to the methyl rotation itself, since in this case methyl rotation is parallel with the CSA tensor. In this case, we should instead calculate the experimental sensitivities by excluding relaxation from the CSA entirely.

Unfortunately, we cannot choose to only fit the methyl rotation or only fit the remaining internal motion– we have to do both together. As mentioned before, this is not a concern for low-field relaxometry experiments, which are not particularly sensitive to fast methyl rotation, and furthermore the CSA interaction is proportional to the magnetic fields and thus is much smaller than the dipole-dipole interaction at low fields. It is also not a

problem for R_2, which is dominated by $J_{CSA}(0)$ and so is also not particularly sensitive to fast methyl rotation. Finally, NOE relaxation rates do not depend on the CSA at all. Then, high-field R_1 experiments present the greatest challenge. In SI Fig. 2, we then plot the sensitivity of ^{13}C R_1 at 950 MHz, with $\Delta\sigma = 75$ ppm and $\Delta\sigma = 0$ ppm, where the former is the correct sensitivity for fitting internal motion excluding methyl rotation, and the latter is the correct sensitivity for fitting the methyl rotation itself.

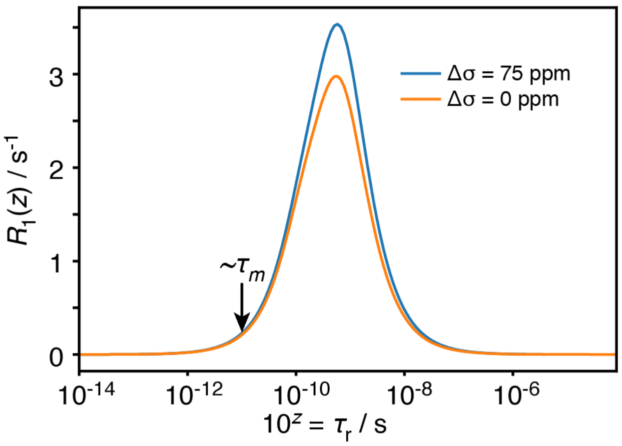

SI Fig. 2. ^{13}C R_1 at 950 MHz as a function of correlation time, calculated using only dipole couplings and using dipole couplings and a CSA scaled to 3 times the desired value (75 ppm instead of 25 ppm). 10 ps is indicated on the plot, the approximate time constant of methyl rotation (Cousin et al. 2018).

While the two functions are clearly different, if we consider the sensitivity near the correlation time of methyl rotation (previously estimated by Cousin et al.(Cousin et al. 2018), near 10 ps), we find that the two functions are nearly converged. For this reason, we simply calculate sensitivities with $\Delta\sigma = 75$ ppm, which is the correct function when considering motions other than rotation, and is nearly correct for the methyl rotation itself.

3. Selecting the number of detectors

We calculate the median error for each detector as a function of the number of detectors using the full experimental data set or the high-field data set. We also report the inverse of the smallest singular value obtained during optimization (according to the procedure previously described (Smith et al. 2019)). This term makes the biggest contribution to the error, $\sigma(\rho_n^{(\theta,S)})$, of the resulting detector responses, which we see in Table S1 and Table S2, where the median errors grow with increasing values of the inverse of the smallest singular value ($[\Sigma]_{n,n}^{-1}$).

Although we can analyse our data with varying number of detectors, a reasonable rule is that the standard deviation should be less than the value for that detector's response

to remain significant (yielding a 68% confidence interval, i.e. 1σ, that the detectors are non-zero). For the full data set analysed with 7 detectors, $\rho_7^{(\theta,S)}$ has a median value of 1.2×10^{-2}, whereas the standard deviation in Table S1 is 1.3×10^{-2}, exceeding the median. However, for analysis with 6 detectors, the smallest median detector response is for $\rho_6^{(\theta,S)}$, having a value of 1.3×10^{-2} and standard deviation of 1.1×10^{-2}. Therefore, we analyse the full data set with 6 detectors. On the other hand, we analyze the high-field data set with 5 detectors in the main text (Figure 2), and with 6 detectors in SI Fig. 3 for comparison.

Note that in Figure 1, we use varying numbers of detectors, based on a threshold value, and we also indicate a threshold in Figure 3. This value was chosen to be $[\Sigma]_{n,n}^{-1} < 1\times10^{-1}$, since this value is slightly larger than the largest singular value when analysing the full data set with 6 detectors.

Table S1. Median standard deviations and maximum inverse singular values for all data

# of detectors	$[\Sigma]_{n,n}^{-1}$	$\sigma(\rho_1^{(\theta,S)})$	$\sigma(\rho_2^{(\theta,S)})$	$\sigma(\rho_3^{(\theta,S)})$	$\sigma(\rho_4^{(\theta,S)})$	$\sigma(\rho_5^{(\theta,S)})$	$\sigma(\rho_6^{(\theta,S)})$	$\sigma(\rho_7^{(\theta,S)})$
1	4.36x10^{-4}	3.95x10^{-4}	–	–	–	–	–	–
2	1.10x10^{-3}	5.62x10^{-4}	6.09x10^{-4}	–	–	–	–	–
3	3.28x10^{-3}	9.13x10^{-4}	1.11x10^{-3}	9.25x10^{-4}	–	–	–	–
4	1.08x10^{-2}	1.12x10^{-3}	1.53x10^{-3}	1.39x10^{-3}	3.01x10^{-3}	–	–	–
5	4.03x10^{-2}	1.34x10^{-3}	2.90x10^{-3}	5.96x10^{-3}	3.58x10^{-3}	4.02x10^{-3}	–	–
6	6.77x10^{-2}	2.44x10^{-3}	5.93x10^{-3}	7.59x10^{-3}	6.73x10^{-3}	8.80x10^{-3}	1.08x10^{-2}	–
7	2.62x10^{-1}	6.19x10^{-3}	7.62x10^{-3}	1.20x10^{-2}	1.72x10^{-2}	2.42x10^{-2}	1.73x10^{-2}	1.32x10^{-2}

Table S2. Median standard deviations and maximum inverse singular values for high-field data

# of detectors	$[\Sigma]_{n,n}^{-1}$	$\sigma(\rho_1^{(\theta,S)})$	$\sigma(\rho_2^{(\theta,S)})$	$\sigma(\rho_3^{(\theta,S)})$	$\sigma(\rho_4^{(\theta,S)})$	$\sigma(\rho_5^{(\theta,S)})$	$\sigma(\rho_6^{(\theta,S)})$	$\sigma(\rho_7^{(\theta,S)})$
1	5.41x10^{-4}	9.74x10^{-4}	–	–	–	–	–	–
2	1.12x10^{-3}	8.96x10^{-4}	7.13x10^{-4}	–	–	–	–	–
3	3.39x10^{-3}	1.23x10^{-3}	1.29x10^{-3}	1.03x10^{-3}	–	–	–	–
4	1.88x10^{-2}	2.14x10^{-3}	1.85x10^{-3}	3.40x10^{-3}	4.71x10^{-3}	–	–	–
5	4.85x10^{-2}	2.75x10^{-3}	5.37x10^{-3}	7.38x10^{-3}	5.20x10^{-3}	6.35x10^{-3}	–	–
6	2.08x10^{-1}	1.08x10^{-2}	6.67x10^{-3}	1.25x10^{-2}	1.91x10^{-2}	2.22x10^{-2}	1.82x10^{-2}	–
7	7.40x10^{-1}	2.14x10^{-2}	1.89x10^{-2}	4.50x10^{-2}	4.78x10^{-2}	4.71x10^{-2}	4.81x10^{-2}	5.18x10^{-2}

4. Additional fits

For additional comparison to the fit of the full relaxometry data set in Fig. 4A, we also fit the high-field data only using 6 detectors. The resulting data fit is found in SI Fig. 7. We see that the six detectors cover a narrower range of correlation times than when processing the full relaxometry data set, and furthermore, exhibit much higher standard deviations for the detector responses at long correlation times.

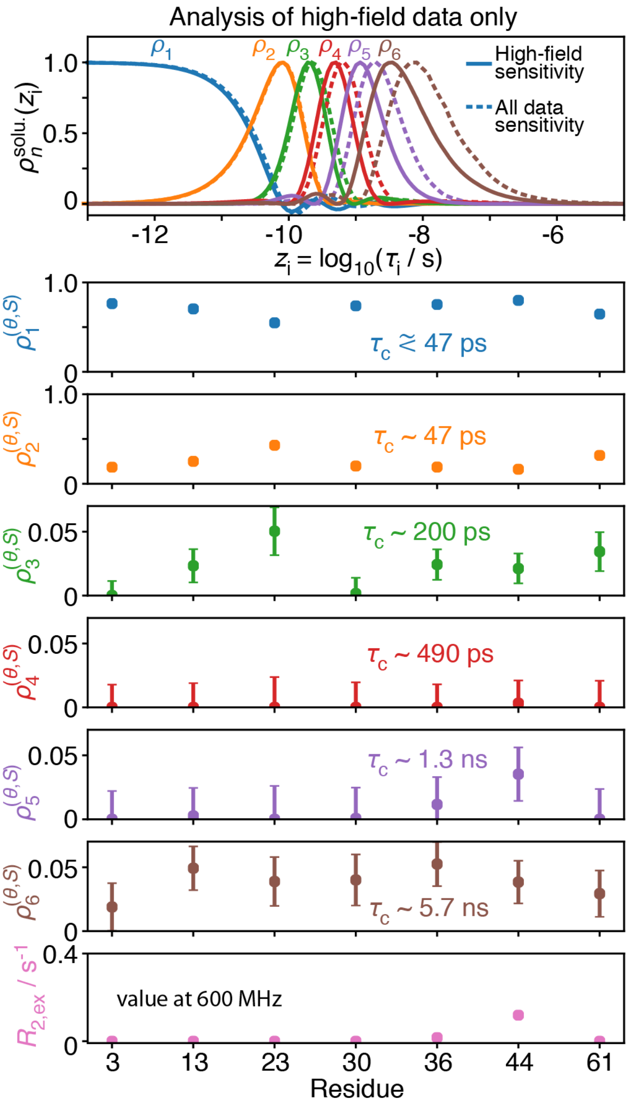

SI Fig. 3. Detector analysis of isoleucine methyl dynamics in ubiquitin, for all 7 Ile in the molecule, using high-field data and 6 detectors (^{13}C relaxation for a $^{13}CD_2H$ group). Residue-specific detector responses are plotted with sensitivities shown at the top (analysis performed assuming τ_r =5.03 ns). For comparison, the sensitivities obtained with the full relaxometry data set are shown with dotted lines in the top plot (same as those shown in Figure 2A). Data fits are found in SI Fig. 7 and Table S3–Table S5.

We also fit only the relaxometry data (20 R_1 experiments between 13.5 and 170 MHz). This is processed with two detectors, according to the threshold established in SI Section 3. We find that relaxometry data alone does not fully separate the slow motions, and rather the combination of high-field and relaxometry is required to provide sufficient resolution to fully characterize the nanosecond regime motion, although we do find that the detector

responses are consistent with the presence of nanosecond motion, as seen in main text Fig. 4. Note that we also lose timescale resolution for the fast methyl rotation, where ρ_1 gives a nearly uniform detector response, as expected, since the order parameter for methyl rotation should be fixed at ~$(1–S^2)$=8/9 (the median detector response of 0.91 is slightly higher than expected for a tetrahedral geometry, consistent with an angle of 111°, as previously shown (Ottiger et al. 1999)).

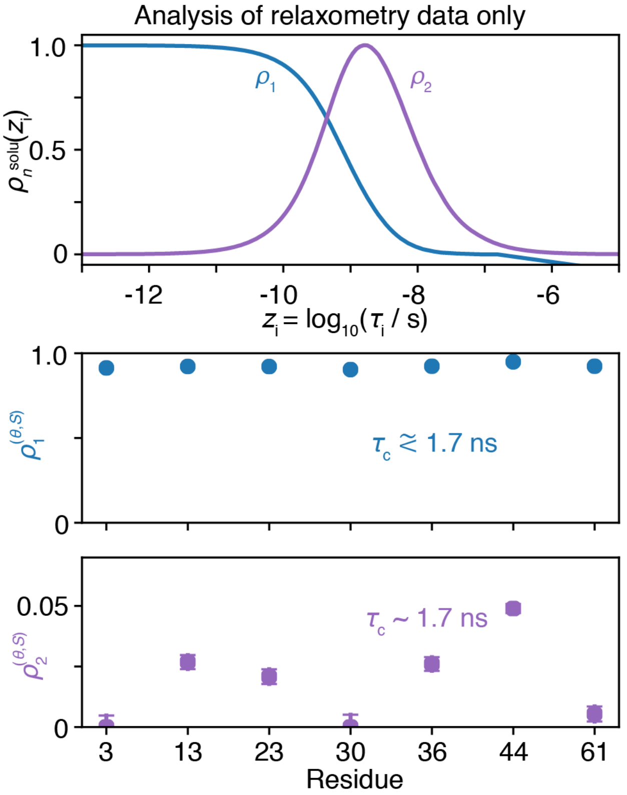

SI Fig. 4. Detector analysis of isoleucine methyl dynamics in ubiquitin, for all 7 Ile in the molecule, using high-relaxometry data only and 2 detectors. Residue-specific detector responses are plotted with sensitivities shown at the top (analysis performed assuming τ_r =5.03 ns).

Experimental data and fitted relaxation rates

Table S3. Nuclear Overhauser Effect Cross-Relaxation rate constants (Cousin et al. 2018)

σ_{HC} / s	3	13	23	30	36	44	61
950 MHz	0.0788	0.1130	0.1976	0.0851	0.0872	0.0772	0.1440
std. dev.	0.0008	0.0011	0.0020	0.0009	0.0009	0.0008	0.0014
Fit(HF, n=5)*	0.0786	0.1131	0.1962	0.0851	0.0872	0.0769	0.1440
Fit (HF, n=6)*	0.0788	0.1131	0.1960	0.0852	0.0873	0.0768	0.1440
Fit (all data)	0.0785	0.1130	0.1952	0.0849	0.0871	0.0767	0.1434
800 MHz	0.0813	0.1185	0.2029	0.0879	0.0921	0.0789	0.1515
std. dev.	0.0017	0.0021	0.0035	0.0019	0.0018	0.0018	0.0027
Fit (HF, n=5)*	0.0801	0.1174	0.2039	0.0870	0.0916	0.0809	0.1497
Fit (HF, n=6)*	0.0796	0.1171	0.2037	0.0865	0.0914	0.0809	0.1495
Fit (all data)	0.0799	0.1166	0.2048	0.0867	0.0913	0.0809	0.1502
600 MHz	0.0786	0.1206	0.2202	0.0859	0.0965	0.0876	0.1557
std. dev.	0.0022	0.0027	0.0043	0.0024	0.0024	0.0024	0.0033
Fit (HF, n=5)*	0.0802	0.1206	0.2083	0.0872	0.0971	0.0872	0.1535
Fit (HF, n=6)*	0.0794	0.1212	0.2107	0.0870	0.0971	0.0872	0.1547
Fit (all data)	0.0800	0.1186	0.2137	0.0872	0.0962	0.0878	0.1568
400 MHz	0.0769	0.1250	0.2291	0.0911	0.1081	0.1096	0.1619
std. dev.	0.0087	0.0111	0.0174	0.0105	0.0101	0.0100	0.0130
Fit (HF, n=5)*	0.0790	0.1206	0.2029	0.0861	0.1037	0.0992	0.1519
Fit (HF, n=6)*	0.0801	0.1259	0.2144	0.0893	0.1057	0.0986	0.1584
Fit (all data)	0.0796	0.1187	0.2164	0.0877	0.1024	0.1006	0.1598

**n* refers to the number of detectors used, where *n*=5 is used in Fig. 4 and SI Fig. 6, and *n*=6 is used in SI Fig. 3 and SI Fig. 7.

Table S4. High field R_1 relaxation rate constants (Cousin et al. 2018)

R_1 / s	3	13	23	30	36	44	61
950 MHz	0.2520	0.3567	0.5433	0.2907	0.3212	0.3092	0.4197
std. dev.	0.0025	0.0036	0.0054	0.0029	0.0032	0.0031	0.0042
Fit(HF, n=5)*	0.2531	0.3590	0.5499	0.2901	0.3218	0.3099	0.4224
Fit (HF, n=6)*	0.2534	0.3605	0.5525	0.2909	0.3227	0.3098	0.4235
Fit (all data)	0.2540	0.3617	0.5539	0.2917	0.3237	0.3119	0.4255
800 MHz	0.2745	0.3886	0.5613	0.3196	0.3561	0.3389	0.4421
std. dev.	0.0038	0.0033	0.0048	0.0039	0.0031	0.0036	0.0040
Fit (HF, n=5)*	0.2765	0.3878	0.5739	0.3217	0.3557	0.3395	0.4459
Fit (HF, n=6)*	0.2754	0.3865	0.5714	0.3203	0.3552	0.3397	0.4440
Fit (all data)	0.2769	0.3897	0.5731	0.3218	0.3570	0.3403	0.4460
600 MHz	0.3424	0.4591	0.6407	0.4041	0.4392	0.4111	0.5088
std. dev.	0.0063	0.0054	0.0058	0.0065	0.0056	0.0059	0.0055
Fit (HF, n=5)*	0.3373	0.4582	0.6285	0.4017	0.4387	0.4072	0.5028
Fit (HF, n=6)*	0.3374	0.4573	0.6267	0.4011	0.4379	0.4074	0.5022
Fit (all data)	0.3380	0.4572	0.6203	0.3987	0.4356	0.4025	0.4967
400 MHz	0.4859	0.6157	0.7364	0.5718	0.6175	0.5246	0.6199
std. dev.	0.0351	0.0311	0.0317	0.0377	0.0325	0.0298	0.0314
Fit (HF, n=5)*	0.4944	0.6217	0.7484	0.5986	0.6298	0.5480	0.6380
Fit (HF, n=6)*	0.5114	0.6437	0.7874	0.6198	0.6383	0.5451	0.6658
Fit (all data)	0.5008	0.6133	0.7399	0.5931	0.6061	0.5222	0.6215

**n* refers to the number of detectors used, where *n*=5 is used in Fig. 4 and SI Fig. 6, and *n*=6 is used in SI Fig. 3 and SI Fig. 7.

Table S5. R_2 relaxation rate constants (Cousin et al. 2018)

R_2 / s	3	13	23	30	36	44	61
950 MHz	2.1834	1.7573	1.9007	2.3050	1.8373	1.7740	2.0381
std. dev.	0.0218	0.0176	0.0190	0.0231	0.0184	0.0180	0.0204
Fit(HF, n=5)*	2.2135	1.7724	1.9572	2.3463	1.8335	1.7757	2.0441
Fit (HF, n=6)*	2.2122	1.7707	1.9531	2.3447	1.8341	1.7756	2.0413
Fit (all data)	2.2134	1.7736	1.9659	2.3531	1.8335	1.7777	2.0450
800 MHz	2.1000	1.7090	1.8794	2.2537	1.7328	1.5140	1.9443
std. dev.	0.0206	0.0154	0.0148	0.0203	0.0161	0.0480	0.0152
Fit (HF, n=5)*	2.1080	1.7029	1.8822	2.2402	1.7371	1.5123	1.9576
Fit (HF, n=6)*	2.1066	1.7008	1.8778	2.2382	1.7365	1.5126	1.9547
Fit (all data)	2.1080	1.7032	1.8891	2.2457	1.7382	1.5137	1.9573
600 MHz	2.0415	1.6600	1.8434	2.1698	1.6654	1.2570	1.8936
std. dev.	0.0171	0.0128	0.0117	0.0166	0.0129	0.0130	0.0125
Fit (HF, n=5)*	2.0196	1.6545	1.8189	2.1578	1.6657	1.2542	1.8855
Fit (HF, n=6)*	2.0211	1.6566	1.8224	2.1596	1.6656	1.2543	1.8880
Fit (all data)	2.0206	1.6524	1.8248	2.1617	1.6646	1.2516	1.8829
400 MHz	2.2231	1.6685	1.8956	2.2922	1.7485	1.0770	2.0531
std. dev.	0.1001	0.0756	0.0712	0.0964	0.0766	0.0700	0.0747
Fit (HF, n=5)*	2.0377	1.6975	1.8293	2.1969	1.7053	1.1348	1.9003
Fit (HF, n=6)*	2.0529	1.7192	1.8677	2.2170	1.7129	1.1326	1.9266
Fit (all data)	2.0438	1.6917	1.8407	2.2009	1.6911	1.1181	1.8936

**n* refers to the number of detectors used, where *n*=5 is used in Fig. 4 and SI Fig. 6, and *n*=6 is used in SI Fig. 3 and SI Fig. 7.

Table S6. Corrected relaxometry relaxation rate constants (Cousin et al. 2018)

R_1 / s	3	13	23	30	36	44	61
389.98 MHz	0.6357	0.6750	0.8105	0.6768	0.6535	0.5668	0.6590
std. dev.	0.0280	0.0223	0.0270	0.0275	0.0217	0.0230	0.0241
Fit (all data)	0.5155	0.6265	0.7508	0.6102	0.6198	0.5312	0.6326
170.42 MHz	1.3996	1.4535	1.3844	1.5706	1.3511	0.9245	1.3810
std. dev.	0.0415	0.0312	0.0319	0.0442	0.0298	0.0232	0.0310
Fit (all data)	1.3918	1.3390	1.4138	1.5835	1.3199	0.9217	1.3135
144.73 MHz	1.6139	1.4074	1.5352	1.8339	1.4510	1.0495	1.4536
std. dev.	0.0578	0.0405	0.0444	0.0607	0.0409	0.0346	0.0428
Fit (all data)	1.6649	1.5505	1.6178	1.8793	1.5272	1.0262	1.5329
127.30 MHz	1.7932	1.6658	1.7000	2.0096	1.6750	1.0929	1.6852
std. dev.	0.0597	0.0426	0.0443	0.0641	0.0418	0.0328	0.0440
Fit (all data)	1.8989	1.7301	1.7903	2.1317	1.7049	1.1140	1.7221
112.32 MHz	2.0303	1.8751	1.9054	2.2500	1.8704	1.1730	1.8607
std. dev.	0.0712	0.0487	0.0524	0.0753	0.0487	0.0359	0.0515
Fit (all data)	2.1409	1.9146	1.9669	2.3920	1.8888	1.2033	1.9184
93.19 MHz	2.6382	2.2632	2.3235	3.0275	2.1595	1.3456	2.1002
std. dev.	0.0863	0.0565	0.0590	0.0910	0.0556	0.0423	0.0556
Fit (all data)	2.5229	2.2031	2.2432	2.8017	2.1776	1.3405	2.2281
86.15 MHz	2.8512	2.4557	2.3957	3.2027	2.4415	1.4341	2.3321
std. dev.	0.1268	0.0741	0.0781	0.1409	0.0733	0.0442	0.0764
Fit (all data)	2.6902	2.3283	2.3638	2.9808	2.3028	1.3987	2.3634
78.34 MHz	2.8788	2.6231	2.5231	3.2007	2.3430	1.4948	2.6162
std. dev.	0.1221	0.0766	0.0784	0.1330	0.0718	0.0465	0.0793
Fit (all data)	2.8976	2.4823	2.5131	3.2022	2.4563	1.4689	2.5307
62.21 MHz	3.6432	2.7901	2.9927	3.5943	2.9030	1.6806	2.9835
std. dev.	0.1525	0.0855	0.0930	0.1477	0.0891	0.0563	0.0953

Fit (all data)	3.4236	2.8662	2.8931	3.7613	2.8348	1.6353	2.9528
53.46 MHz	3.7823	3.1106	3.0024	4.3483	3.0772	1.7532	3.1878
std. dev.	0.1814	0.1066	0.1044	0.1952	0.1068	0.0691	0.1116
Fit (all data)	3.7887	3.1267	3.1587	4.1466	3.0874	1.7408	3.2449
45.81 MHz	4.0378	3.3221	3.4250	4.5860	3.3625	1.8338	3.5543
std. dev.	0.1689	0.0926	0.0990	0.1793	0.0924	0.0561	0.1036
Fit (all data)	4.1757	3.3976	3.4414	4.5524	3.3469	1.8448	3.5551
33.60 MHz	5.1943	3.9036	4.2082	5.4937	4.0225	1.9022	4.3136
std. dev.	0.2707	0.1327	0.1468	0.2917	0.1342	0.0686	0.1507
Fit (all data)	4.9886	3.9519	4.0339	5.3954	3.8749	2.0464	4.2140
30.32 MHz	5.2452	4.0841	4.2223	5.8072	3.9862	1.9760	4.3690
std. dev.	0.2899	0.1350	0.1438	0.2967	0.1359	0.0758	0.1488
Fit (all data)	5.2613	4.1345	4.2307	5.6757	4.0500	2.1116	4.4384
27.63 MHz	5.2076	4.2507	4.5373	5.6826	4.1927	1.9931	4.4288
std. dev.	0.2037	0.1049	0.1187	0.2210	0.1065	0.0496	0.1189
Fit (all data)	5.5053	4.2969	4.4055	5.9255	4.2069	2.1697	4.6408
27.63 MHz	5.2565	4.0302	4.4134	5.4090	4.1115	2.1018	4.1965
std. dev.	0.2653	0.1315	0.1493	0.2627	0.1330	0.0736	0.1505
Fit (all data)	5.5053	4.2969	4.4055	5.9256	4.2069	2.1697	4.6408
23.89 MHz	6.0764	4.3274	4.9319	6.4097	4.7690	2.1793	5.2090
std. dev.	0.3833	0.1683	0.1911	0.3775	0.1782	0.0922	0.2082
Fit (all data)	5.8745	4.5409	4.6670	6.3020	4.4455	2.2583	4.9502
18.75 MHz	6.6194	5.0581	5.1289	7.9194	4.7781	2.3791	5.8414
std. dev.	0.2770	0.1462	0.1483	0.3152	0.1418	0.0802	0.1678
Fit (all data)	6.4334	4.9078	5.0550	6.8687	4.8121	2.3954	5.4257
14.03 MHz	6.9842	5.4165	5.3494	7.9907	5.0494	2.6422	5.6521
std. dev.	0.3721	0.1864	0.1893	0.3991	0.1791	0.0907	0.2051
Fit (all data)	6.9720	5.2596	5.4190	7.4119	5.1737	2.5330	5.8922
14.23 MHz	7.3603	5.4475	5.6921	8.2305	5.2381	2.6349	6.0103
std. dev.	0.3810	0.1904	0.2023	0.4135	0.1848	0.0947	0.2221
Fit (all data)	6.9496	5.2450	5.4041	7.3894	5.1585	2.5272	5.8727
14.18 MHz	7.3358	5.6000	5.3421	7.8312	5.2677	2.8796	6.2468
std. dev.	0.3298	0.1799	0.1635	0.3499	0.1773	0.0896	0.2048
Fit (all data)	6.9554	5.2487	5.4079	7.3952	5.1624	2.5287	5.8777
13.52 MHz	6.4858	5.5726	5.9062	7.2461	5.2470	2.4186	5.6780
std. dev.	0.3840	0.1884	0.2155	0.4216	0.1765	0.0820	0.2226
Fit (all data)	7.0304	5.2976	5.4579	7.4706	5.2134	2.5483	5.9433

Table S7. Relaxation rate constants acquired with the two-field system (Kaderavek et al. 2019)

R_2 / s	**3**	**13**	**23**	**30**	**36**	**44**	**61**
R_1 (*14 MHz*)	7.3000	5.0100	5.5500	7.9800	5.0100	2.7000	6.3500
std. dev.	0.4800	0.2300	0.2300	0.5700	0.2300	0.1000	0.2800
Fit (all data)	6.9758	5.2620	5.4215	7.4157	5.1762	2.5340	5.8956
R_2 (*14 MHz*)	7.2900	5.1200	5.7200	7.3400	5.2300	2.7600	6.4200
std. dev.	0.3400	0.1600	0.1600	0.3400	0.1800	0.0800	0.1900
Fit (all data)	7.1631	5.3840	5.5445	7.6037	5.3056	2.5844	6.0606

These experiments are performed with the two-field system (Kaderavek et al. 2019), which allows pulses to be applied at low field (14 MHz), therefore yielding the correct relaxation rates without using ICARUS correction. These rates are only fit in combination with the relaxometry data set.

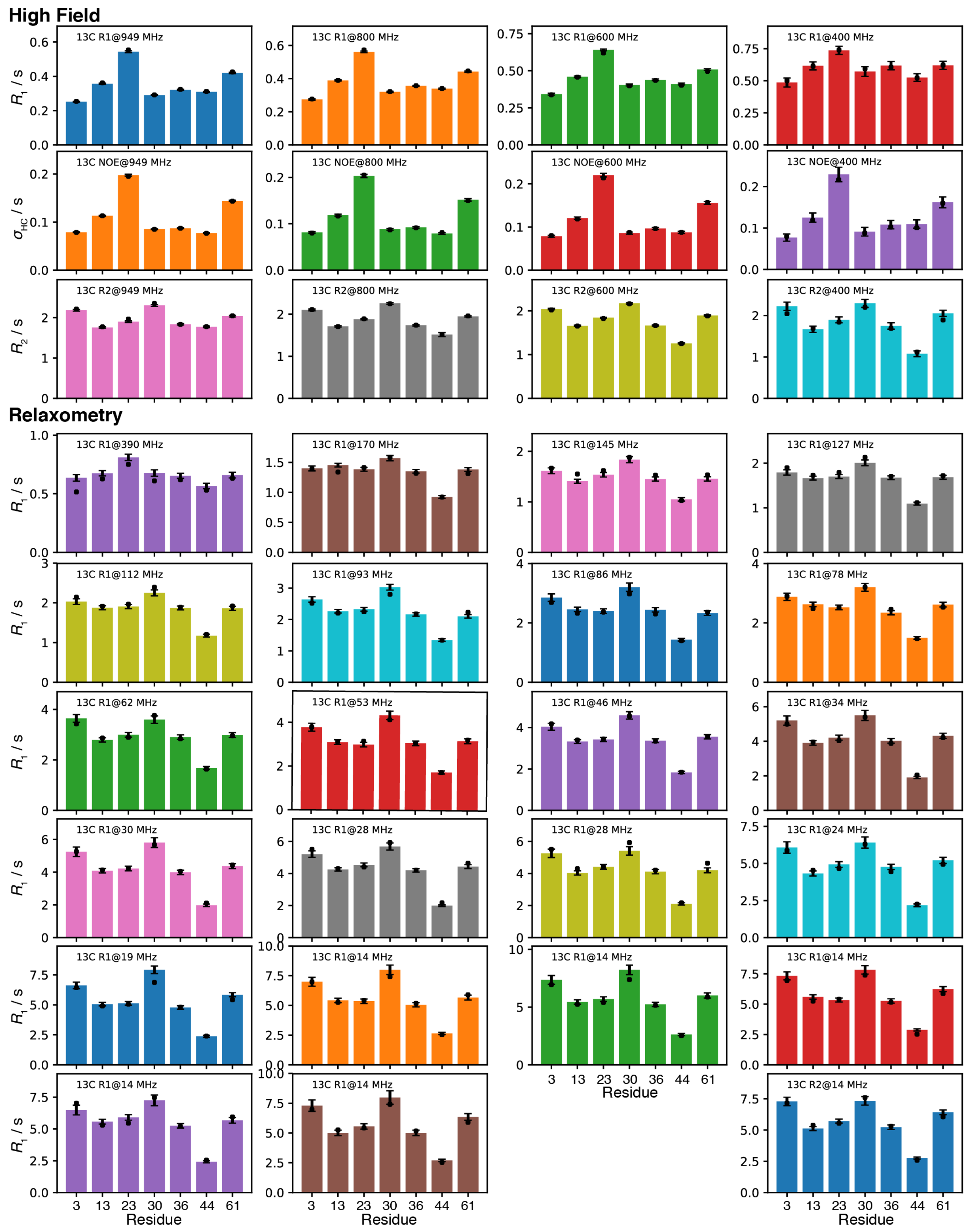

SI Fig. 5. Fit of full experimental data set using the detector analysis. Bars show the measured rates (relaxometry data corrected with the ICARUS procedure) as well as the standard deviation of the measurement. Scatter points show the fitted rate constant. Fit corresponds to detector analysis in Figure 3A.

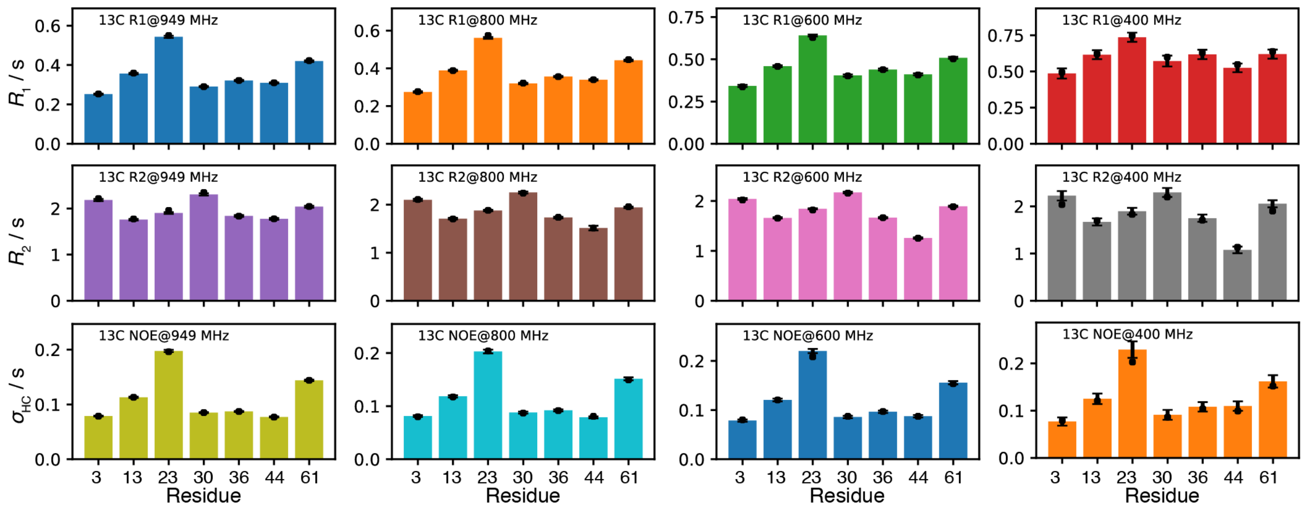

SI Fig. 6. Fit of high-field experimental data set using the detector analysis with 5 detectors. Bars show the measured rates as well as the standard deviation of the measurement. Scatter points show the fitted rate constant. Fit corresponds to the detector analysis in Figure 3B.

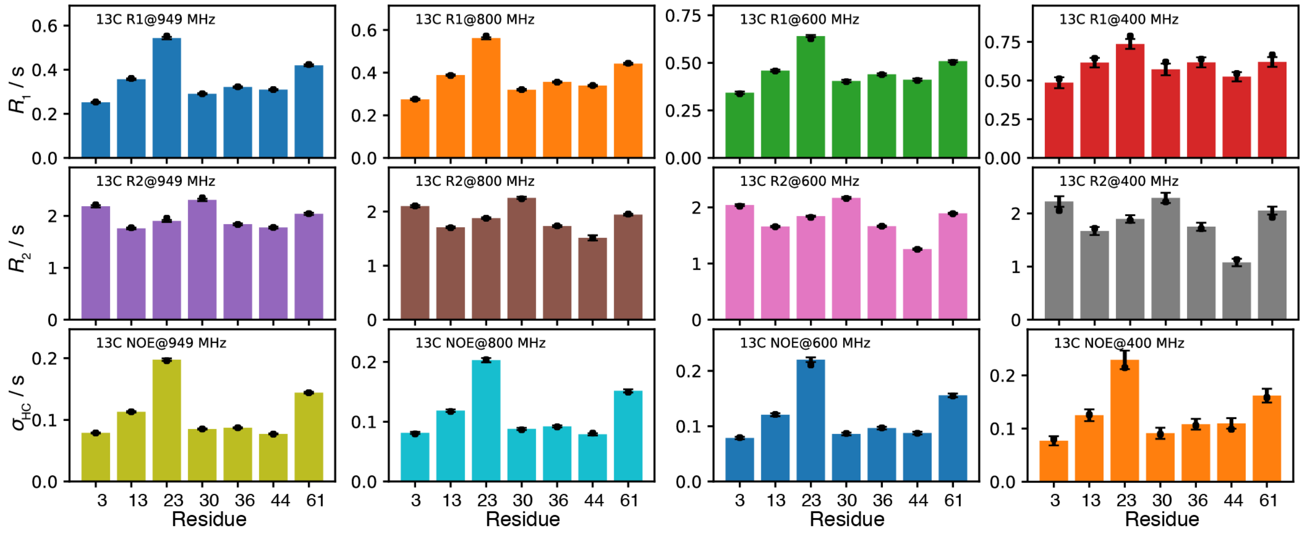

SI Fig. 7. Fit of high-field experimental data set using the detector analysis with 6 detectors. Bars show the measured rates as well as the standard deviation of the measurement. Scatter points show the fitted rate constant. Fit corresponds to the detector analysis in SI Fig. 3.

Table S8: Detection vectors for analysis of all data (see Fig. 4A, SI Fig. 5)

	$\vec{r}_1$ / s^{-1}	$\vec{r}_2$ / s^{-1}	$\vec{r}_3$ / s^{-1}	$\vec{r}_4$ / s^{-1}	$\vec{r}_5$ / s^{-1}	$\vec{r}_6$ / s^{-1}	$\vec{r}_{ex}$ / s^{-1}	R_0
$R_{1,950}$	-0.9346	0.2208	1.260	2.005	1.160	0.1332	0.000	0.9337
$R_{1,800}$	-1.242	-0.03748	1.181	2.072	1.474	0.1818	0.000	1.240
$R_{1,600}$	-2.033	-0.7109	0.9391	2.017	2.060	0.3372	0.000	2.031
$R_{1,400}$	-4.084	-2.433	0.05539	1.372	2.665	0.7958	0.000	4.089
$R_{1,597}$	-4.268	-2.584	-0.03585	1.301	2.679	0.8353	0.000	4.273
$R_{1,597}$	-15.32	-11.35	-6.513	-4.178	-0.1245	1.696	0.000	15.33
$R_{1,400}$	-18.88	-14.14	-8.660	-6.249	-1.474	1.461	0.000	18.89
$R_{1,400}$	-21.95	-16.56	-10.50	-8.133	-2.676	1.145	0.000	21.96
$R_{1,389}$	-25.16	-19.09	-12.40	-10.17	-3.952	0.7478	0.000	25.17
$R_{1,170}$	-30.25	-23.13	-15.38	-13.54	-6.044	0.05644	0.000	30.27
$R_{1,144}$	-32.49	-24.90	-16.67	-15.05	-7.000	-0.2478	0.000	32.51
$R_{1,127}$	-35.26	-27.10	-18.28	-16.94	-8.230	-0.6140	0.000	35.28
$R_{1,112}$	-42.29	-32.68	-22.34	-21.77	-11.62	-1.473	0.000	42.32
$R_{1,93}$	-47.16	-36.54	-25.18	-25.10	-14.25	-2.044	0.000	47.20
$R_{1,86}$	-52.35	-40.63	-28.22	-28.59	-17.27	-2.704	0.000	52.39
$R_{1,78}$	-63.37	-49.29	-34.74	-35.87	-24.27	-4.600	0.000	63.42
$R_{1,62}$	-67.13	-52.24	-36.97	-38.32	-26.76	-5.464	0.000	67.18
$R_{1,53}$	-70.53	-54.90	-38.98	-40.53	-29.03	-6.354	0.000	70.57
$R_{1,45}$	-70.53	-54.90	-38.98	-40.53	-29.03	-6.354	0.000	70.57
$R_{1,33}$	-75.72	-58.97	-42.05	-43.92	-32.51	-7.921	0.000	75.77
$R_{1,30}$	-83.72	-65.26	-46.75	-49.17	-37.85	-10.81	0.000	83.77
$R_{1,27}$	-91.59	-71.48	-51.32	-54.39	-43.01	-14.20	0.000	91.65
$R_{1,27}$	-91.26	-71.22	-51.13	-54.17	-42.79	-14.05	0.000	91.32
$R_{1,23}$	-91.35	-71.28	-51.18	-54.23	-42.85	-14.09	0.000	91.41
$R_{1,19}$	-92.46	-72.16	-51.82	-54.97	-43.57	-14.61	0.000	92.52
$R_{1,14}$	-91.65	-71.52	-51.35	-54.43	-43.04	-14.23	0.000	91.71
$R_{1,14}$	-27.67	-20.89	-14.23	-15.10	-12.49	-5.482	2.504	27.69
$R_{1,14}$	-26.26	-19.80	-13.30	-14.05	-11.48	-5.101	1.780	26.27
$R_{1,14}$	-25.08	-18.93	-12.41	-12.97	-10.27	-4.610	1.001	25.10
$R_{1,14}$	-25.32	-19.20	-12.31	-12.46	-9.369	-4.039	0.4449	25.34
$R_{2,950}$	-94.44	-73.74	-52.96	-56.31	-44.83	-15.62	0.0005	94.51
$R_{2,800}$	-0.02169	0.4122	0.3069	0.08739	0.02519	-0.001374	0.000	0.02149
$R_{2,600}$	-0.03123	0.4124	0.4341	0.1223	0.03966	-0.003210	0.000	0.03024
$R_{2,400}$	-0.05430	0.3810	0.6712	0.2488	0.06594	-0.001115	0.000	0.05379
$R_{2,14}$	-0.1191	0.2805	0.9528	0.6518	0.1292	0.01733	0.000	0.1212
$\sigma_{HC,950}$	-0.9346	0.2208	1.260	2.005	1.160	0.1332	0.000	0.9337
$\sigma_{HC,800}$	-1.242	-0.03748	1.181	2.072	1.474	0.1818	0.000	1.240
$\sigma_{HC,600}$	-2.033	-0.7109	0.9391	2.017	2.060	0.3372	0.000	2.031
$\sigma_{HC,400}$	-4.084	-2.433	0.05539	1.372	2.665	0.7958	0.000	4.089

Other parameters: τ_r=5.03 ns, $\Delta\sigma$=*75 ppm, δ_{HC}=43588 Hz, δ_{DC}= 6782 Hz
*$\Delta\sigma$ is set to 3 times the desired value (25 ppm) as discussed in SI Section 2.

Table S9: Detection vectors for analysis at high-field, n=5 (see Fig. 4B, SI Fig. 6)

	$\vec{r}_1$ / s^{-1}	$\vec{r}_2$ / s^{-1}	$\vec{r}_3$ / s^{-1}	$\vec{r}_4$ / s^{-1}	$\vec{r}_5$ / s^{-1}	$\vec{r}_{ex}$ / s^{-1}	R_0
$R_{1,950}$	-0.9339	0.2219	1.280	2.110	0.7085	0.000	0.9337
$R_{1,800}$	-1.242	-0.03890	1.202	2.212	0.9867	0.000	1.240
$R_{1,600}$	-2.033	-0.7317	0.9920	2.180	1.605	0.000	2.031
$R_{1,400}$	-4.076	-2.556	0.2957	1.358	2.668	0.000	4.089
$R_{2,950}$	-27.67	-21.56	-13.58	-15.58	-11.81	2.504	27.69
$R_{2,800}$	-26.26	-20.43	-12.69	-14.49	-10.87	1.780	26.27
$R_{2,600}$	-25.08	-19.54	-11.80	-13.39	-9.674	1.001	25.10
$R_{2,400}$	-25.32	-19.87	-11.56	-13.01	-8.494	0.4449	25.34
$\sigma_{HC,950}$	-0.02163	0.4209	0.2863	0.09040	0.002193	0.000	0.02149
$\sigma_{HC,800}$	-0.03130	0.4222	0.4132	0.1243	0.007239	0.000	0.03024
$\sigma_{HC,600}$	-0.05429	0.3903	0.6568	0.2436	0.01286	0.000	0.05379
$\sigma_{HC,400}$	-0.1181	0.2822	0.9657	0.6308	0.02205	0.000	0.1212

Other parameters: τ_r=5.03 ns, Δσ=*75 ppm, δ_{HC}=43588 Hz, δ_{DC}= 6782 Hz
*Δσ is set to 3 times the desired value (25 ppm) as discussed in SI Section 2.

Table S10: Detection vectors for analysis at high-field, n=6 (see SI Fig. 3, SI Fig. 7)

	$\vec{r}_1$ / s^{-1}	$\vec{r}_2$ / s^{-1}	$\vec{r}_3$ / s^{-1}	$\vec{r}_4$ / s^{-1}	$\vec{r}_5$ / s^{-1}	$\vec{r}_6$ / s^{-1}	$\vec{r}_{ex}$ / s^{-1}	R_0
$R_{1,950}$	-0.9357	0.1371	1.106	1.602	1.580	0.4341	0.000	0.9337
$R_{1,800}$	-1.242	-0.1514	1.079	1.525	1.861	0.5870	0.000	1.240
$R_{1,600}$	-2.033	-0.8737	0.9239	1.260	2.246	0.9781	0.000	2.031
$R_{1,400}$	-4.092	-2.646	0.1566	0.4607	2.214	1.786	0.000	4.089
$R_{2,950}$	-27.67	-22.41	-9.681	-18.08	-8.860	-11.95	5.628	27.69
$R_{2,800}$	-26.26	-21.25	-9.020	-16.94	-8.094	-11.09	4.000	26.27
$R_{2,600}$	-25.08	-20.31	-8.406	-15.77	-7.248	-10.01	2.250	25.10
$R_{2,400}$	-25.33	-20.54	-8.468	-15.25	-6.828	-8.963	1.000	25.34
$\sigma_{HC,950}$	-0.02179	0.3995	0.2902	0.1313	0.04035	0.005320	0.000	0.02149
$\sigma_{HC,800}$	-0.03105	0.3950	0.4093	0.1801	0.06215	0.005641	0.000	0.03024
$\sigma_{HC,600}$	-0.05441	0.3619	0.6109	0.3397	0.1072	0.01519	0.000	0.05379
$\sigma_{HC,400}$	-0.1213	0.2734	0.8010	0.7615	0.2485	0.04994	0.000	0.1212

Other parameters: τ_r=5.03 ns, Δσ=*75 ppm, δ_{HC}=43588 Hz, δ_{DC}= 6782 Hz
*Δσ is set to 3 times the desired value (25 ppm) as discussed in SI Section 2.

5. References

Bolik-Coulon, N., P. Kaderavek, P. Pelupessy, J. N. Dumez, F. Ferrage and S. F. Cousin (2020). Theoretical and computational framework for the analysis of the relaxation properties of arbitrary spin systems. Application to high-resolution relaxometry. J. Magn. Res. 313: 106718.

Charlier, C., S. N. Khan, T. Marquardsen, P. Pelupessy, V. Reiss, D. Sakellariou, G. Bodenhausen, F. Engelke and F. Ferrage (2013). Nanosecond time scale motions in proteins revealed by high-resolution NMR relaxometry. J. Am. Chem. Soc. 135(49): 18665-18672.

Cousin, S. F., P. Kaderavek, N. Bolik-Coulon, Y. Gu, C. Charlier, L. Carlier, L. Bruschweiler-Li, T. Marquardsen, J. M. Tyburn, R. Bruschweiler and F. Ferrage (2018). Time-Resolved Protein Side-Chain Motions Unraveled by High-Resolution Relaxometry and Molecular Dynamics Simulations. J. Am. Chem. Soc. 140(41): 13456-13465.

Kaderavek, P., N. Bolik-Coulon, S. F. Cousin, T. Marquardsen, J. M. Tyburn, J. N. Dumez and F. Ferrage (2019). Protein Dynamics from Accurate Low-Field Site-Specific Longitudinal and Transverse Nuclear Spin Relaxation. J. Phys. Chem. Lett. 10(19): 5917-5922.

Ottiger, M. and A. Bax (1999). How Tetrahedral Are Methyl Groups in Proteins? A Liquid Crystal NMR Study. J. Am. Chem. Soc. 121: 4690-4695.

Smith, A. A., M. Ernst and B. H. Meier (2018). Optimized "detectors" for dynamics analysis in solid-state NMR. J. Chem. Phys. 148(4): 045104.

Smith, A. A., M. Ernst, B. H. Meier and F. Ferrage (2019). Reducing bias in the analysis of solution-state NMR data with dynamics detectors. J. Chem. Phys. 151(3): 034102.
